# Supplementary material for: Evaluation of non-invasive continuous physiological monitoring devices for neonates in Nairobi, Kenya: a research protocol
Source: BMJ Open. 2020 Apr 12;10(4):e035184. doi: 10.1136/bmjopen-2019-035184 (PMC7200030; doi:10.1136/bmjopen-2019-035184)
Supplement: Supplementary data [file bmjopen-2019-035184supp001.pdf]

1 **Appendix I: Schedule of study procedures and evaluations**

Activity	Screening	Enrollment	Observation	Discharge
Eligibility assessment	X			
Informed consent and comprehension checklist	X			
Assign participant ID	X			
Demographics	X	X		
Medical history		X	X	X
Maternal pregnancy history		X		
Medications use		X		
Placement of reference and/or investigational device(s)		X	X	
Collection of vital signs		X	X	X
Video tape recording and/or photographs			X	

Ginsburg AS, *et al. BMJ Open* 2020; 10:e035184. doi: 10.1136/bmjopen-2019-035184

Track clinical care and non-study activities			X	X
Safety assessment			X	
End of study questions				X
Removal of investigational and/or reference device(s)				X

2

Ginsburg AS, *et al. BMJ Open* 2020; 10:e035184. doi: 10.1136/bmjopen-2019-035184
