# Supplementary material for: Evaluation of non-invasive continuous physiological monitoring devices for neonates in Nairobi, Kenya: a research protocol
Source: BMJ Open. 2020 Apr 12;10(4):e035184. doi: 10.1136/bmjopen-2019-035184 (PMC7200030; doi:10.1136/bmjopen-2019-035184)
Supplement: Author's manuscript [file bmjopen-2019-035184.draft_revisions.pdf]

BMJ Open is committed to open peer review. As part of this commitment we make the peer review history of every article we publish publicly available.

When an article is published we post the peer reviewers' comments and the authors' responses online. We also post the versions of the paper that were used during peer review. These are the versions that the peer review comments apply to.

The versions of the paper that follow are the versions that were submitted during the peer review process. They are not the versions of record or the final published versions. They should not be cited or distributed as the published version of this manuscript.

BMJ Open is an open access journal and the full, final, typeset and author-corrected version of record of the manuscript is available on our site with no access controls, subscription charges or pay-per-view fees (http://bmjopen.bmj.com).

If you have any questions on BMJ Open's open peer review process please email info.bmjopen@bmj.com

BMJ Open

Evaluation of noninvasive continuous physiological monitoring devices for neonates in Nairobi, Kenya: A research protocol

Journal:	*BMJ Open*
Manuscript ID	bmjopen-2019-035184
Article Type:	Protocol
Date Submitted by the Author:	21-Oct-2019

Complete List of Authors:	ginsburg, amy sarah; Save the Children Federation Inc<br>Nkwopara, Evangelyn; Save the Children Federation Inc<br>Macharia, William; The Aga Khan University, Paediatrics<br>Ochieng, Roseline; Aga Khan University - Kenya, Faculty of Health Sciences<br>Waiyego, Mary ; Pumwani Maternity Hospital<br>Zhou, Guohai; Brigham and Women's Hospital<br>Karasik, Roman; EarlySense<br>Xu, Shuai; Sibel Inc.; Northwestern University<br>Ansermino, J. Mark; The University of British Columbia

Keywords:	NEONATOLOGY, Physiology < BASIC SCIENCES, Quality in health care < HEALTH SERVICES ADMINISTRATION & MANAGEMENT

SCHOLARONE™
Manuscripts

I, the Submitting Author has the right to grant and does grant on behalf of all authors of the Work (as defined in the below author licence), an exclusive licence and/or a non-exclusive licence for contributions from authors who are: i) UK Crown employees; ii) where BMJ has agreed a CC-BY licence shall apply, and/or iii) in accordance with the terms applicable for US Federal Government officers or employees acting as part of their official duties; on a worldwide, perpetual, irrevocable, royalty-free basis to BMJ Publishing Group Ltd ("BMJ") its licensees and where the relevant Journal is co-owned by BMJ to the co-owners of the Journal, to publish the Work in this journal and any other BMJ products and to exploit all rights, as set out in our licence.

The Submitting Author accepts and understands that any supply made under these terms is made by BMJ to the Submitting Author unless you are acting as an employee on behalf of your employer or a postgraduate student of an affiliated institution which is paying any applicable article publishing charge ("APC") for Open Access articles. Where the Submitting Author wishes to make the Work available on an Open Access basis (and intends to pay the relevant APC), the terms of reuse of such Open Access shall be governed by a Creative Commons licence – details of these licences and which Creative Commons licence will apply to this Work are set out in our licence referred to above.

Other than as permitted in any relevant BMJ Author's Self Archiving Policies, I confirm this Work has not been accepted for publication elsewhere, is not being considered for publication elsewhere and does not duplicate material already published. I confirm all authors consent to publication of this Work and authorise the granting of this licence.

For peer review only

Title: Evaluation of noninvasive continuous physiological monitoring devices for neonates in Nairobi, Kenya: A research protocol

Authors: Amy Sarah Ginsburg,[1]* Evangelyn Nkwopara,[1] William Macharia,[2] Roseline Ochieng,[2] Mary Waiyego,[3] Guohai Zhou,[4] Roman Karasik,[5] Shuai Xu,[6,7] and J. Mark Ansermino[8]

[1] Save the Children Federation, Inc., Fairfield, Connecticut, United States of America

[2] Aga Khan University, Nairobi, Kenya

[3] Pumwani Maternity Hospital, Nairobi, Kenya

[4] Brigham and Women's Hospital, Harvard Medical School, Boston, United States of America

[5] EarlySense Ltd., Ramat-Gan, Israel

[6] Sibel Inc., Evanston, Illinois, United States of America

[7] Northwestern University, Evanston, Illinois, United States of America

[8] The University of British Columbia, Vancouver, Canada

For peer review only

20 * Corresponding author:

21 Mailing address: Save the Children Federation Inc.,

22 501 King's Highway E, Suite 400

23 Fairfield, CT 06825, USA

24 Phone: 203.221.4000

25 Email: aginsburg@savechildren.org

For peer review only

For peer review only - http://bmjopen.bmj.com/site/about/guidelines.xhtml

ABSTRACT

Introduction: Continuous physiological monitoring devices are often not available for monitoring high-risk neonates in low-resource settings. Easy-to-use, noninvasive, multiparameter, continuous physiological monitoring devices could be instrumental in providing appropriate care and improving outcomes for high-risk neonates in these low-resource settings.

Methods and Analysis: The purpose of this prospective, observational, facility-based evaluation is to provide evidence to establish whether two existing noninvasive, multiparameter, continuous physiological monitoring devices developed by device developers, EarlySense and Sibel, can accurately and reliably measure vital signs in neonates (when compared to verified reference devices). We will also assess the feasibility, usability and acceptability of these devices for use in neonates in low-resource settings in Africa. Up to 500 neonates are enrolled in two phases: 1) a verification and accuracy evaluation phase at Aga Khan University - Nairobi; and 2) a clinical feasibility phase at Pumwani Maternity Hospital in Nairobi, Kenya. Both quantitative and qualitative data are collected and analyzed. Agreement between the investigational and reference devices is determined using *a priori*-defined accuracy thresholds.

Discussion: We hypothesize that the investigational devices are equivalent to the reference devices for each relevant measurement parameter of interest among neonates, and that the investigational devices are feasible, usable and acceptable for use in neonates in low-resource settings in Africa.

For peer review only - http://bmjopen.bmj.com/site/about/guidelines.xhtml

Ethics and Dissemination: This trial was approved by the Aga Khan University Nairobi Research Ethics Committee and the Western Institutional Review Board. We plan to disseminate research results in peer-reviewed journals and international conferences.

ClinicalTrials.gov NCT03920761

Keywords: neonates, continuous physiological monitoring devices

For peer review only

For peer review only - http://bmjopen.bmj.com/site/about/guidelines.xhtml

STRENGTHS AND LIMITATIONS OF THIS STUDY

- This research consists of two phases, a verification and accuracy evaluation phase and a clinical feasibility phase, and evaluation of two novel, investigational, noninvasive, multiparameter, continuous physiological monitoring devices.
- A verification of the reference devices is undertaken prior to initiating the accuracy evaluation of the investigational devices to ensure the reference devices are robustly functional and to confirm their within subject repeatability and accuracy compare to standard clinical measurements for the relevant parameters of interest.
- Reliability information gathered from the reference devices is utilized to determine specific *a priori* Go/No Go criteria for each parameter and each investigational device.
- As with all measurements, there is uncertainty inherent in the measurements from the reference devices.
- Inability to control for the characteristics and conditions of the participating neonates and to standardize the environment and context are both strengths and limitations to interpreting the results.

For peer review only - http://bmjopen.bmj.com/site/about/guidelines.xhtml

INTRODUCTION

In 2017 globally, 47% of all deaths in children under 5 years of age occurred within the first 28 days of life, which translates to a neonatal mortality rate of 18 deaths per 1000 live births or 2.5 million newborn deaths.[1] Sub-Saharan Africa bears the greatest burden of neonatal mortality with an estimated 1 million newborn deaths in 2017. Further efforts, especially in African countries, are needed to push progress towards achieving the Sustainable Development Goal (SDG) target of reducing global neonatal mortality to 12 deaths per 1000 live births by 2030.[2] Without accelerated improvements, it is projected that 1.8 million neonates will die in 2030.[3] Innovations in neonatal care, particularly technologies that allow for early detection and intervention for major morbidities, hold great promise in helping to reduce current and projected neonatal mortality rates.

Multiparameter continuous physiological monitoring devices could be instrumental in identifying neonates at risk. We can then direct care provided for a neonate through automatic interpretations of vital signs that help identify critical events and determine if treatment is sufficient or insufficient, ultimately improving newborn outcomes.[4,5] These devices would be most useful in low-resource settings where the need for such technologies is greatest. While continuous physiological monitoring is standard of care in high-resource settings, the devices are expensive and require specialized training to operate, making them unsuitable for application in low-resource settings. To address these barriers, it is necessary to explore how these technologies can be adapted and/or optimized for use in low-resource settings. Ideally, the devices should be low cost, operator-independent, noninvasive and highly efficient in diagnostic performance and operator workload. This requires development of a robust testing platform that appropriately mimics conditions common in

For peer review only - http://bmjopen.bmj.com/site/about/guidelines.xhtml

African newborn or neonatal intensive care units that would allow these type of technologies to be evaluated for feasibility and performance.

The Evaluation of Technologies for Neonates in Africa (ETNA) project was conceived with the goal of advancing and supporting development, as well as evaluation, of select devices for use in neonates. By establishing a testing platform in an African site, and working collaboratively with partners with expertise in device development and evaluation and neonatal and child health, the project seeks to boost development and optimization of promising newborn care devices that could be applied in low-resource settings in Africa. The purpose of this initial research is to produce evidence regarding the performance of two existing noninvasive, multiparameter, continuous physiological monitoring devices developed by device developers, EarlySense and Sibel. The intent is to provide evidence to establish whether these investigational devices can accurately and reliably measure vital signs in neonates (when compared to verified reference devices) and to assess the feasibility, usability and acceptability of these devices for use in neonates in a low-resource settings in Africa.

METHODS AND ANALYSIS

Study design and setting

The primary objectives of this prospective, observational, facility-based research are: 1) to assess agreement between repeat observations by the investigational device and the reference device for each relevant measurement parameter of interest based on *a priori*-determined accuracy threshold among neonates; 2) to compare clinical event detection

For peer review only - http://bmjopen.bmj.com/site/about/guidelines.xhtml

performance between the investigational device and the reference device; and 3) to determine whether the investigational device is feasible, usable and acceptable to hospital administrators, healthcare providers (HCPs) and caregivers of neonates. Secondary objectives include: 1) assessing diagnostic performance for each relevant measurement parameter of interest based on sensitivity, specificity, positive predictive value, and negative predictive value compared to the reference device; 2) determining the downtime performance of the investigational device; 3) determining the alarm rate (events/hour) and the number of true/false alarms of the investigational device compared to the reference device; 4) determining the delay time between the investigational device and the reference device in true events; and 5) determining the number of adverse device effects (ADEs) and serious adverse events (SAEs) during use of the investigational device.

Taking place in Nairobi, Kenya, this research consists of two phases: 1) a verification and accuracy evaluation phase conducted at Aga Khan University – Nairobi (AKU-N), a private, not-for-profit university teaching hospital with a neonatal intensive care and high dependency units; and 2) a clinical feasibility phase conducted at Pumwani Maternity Hospital (PMH), the largest referral maternity hospital in sub-Saharan Africa with no neonatal intensive care or high dependency units.

Study participants

Up to 500 neonates, corrected age of ≥28 days admitted for routine observation and care at AKU-N and PMH are recruited by trained study staff during routine intake and screening procedures. To avoid potential selection bias, neonates are screened for enrollment in a sequential manner, as much as possible. Trained study staff assess the neonate for all inclusion and exclusion criteria (Table 1). Final eligibility determination is dependent on the results of the medical history, clinical examination, appropriate understanding of the study

For peer review only

For peer review only - http://bmjopen.bmj.com/site/about/guidelines.xhtml

by the caregiver, and completion of the written informed consent process. A neonate may be enrolled to the study more than once as long as they meet the eligibility criteria and the caregiver(s) is willing to have the neonate participate.

For the feasibility, usability and acceptability assessment, hospital administrators and study HCPs are enrolled if they are 18 years or older, involved in or aware of the ETNA study, and have provided written informed consent. Caregivers may be enrolled if they are 18 years or older, have a neonate enrolled in the study, and are willing to participate in a 30-minute in-depth interview as well as direct observation while their neonate is on or attached to the investigational device(s).

Investigational devices

Developed by Israeli-based EarlySense since 2009, the Insight system, released in 2016, is a contact-free monitoring system comprised of a small piezoelectric sensor pad that can be placed under the patient's mattress, and is designed to measure and record a patient's heart rate (HR), respiratory rate (RR), motion, and sleep status. Information from the sensor pad, in combination with EarlySense's artificial intelligence analytics, is transmitted to a monitor to provide alert indications and vital sign trends to HCPs so that they can monitor changes in a patient's condition. Currently in use in hospitals, rehabilitative centers, and nursing homes to measure vital signs in adults and children above 10 kg, the device is modified for use in neonates as part of this study. The adult device received regulatory approval from the United States Federal Drug Administration (FDA) and has a Conformité Européene (CE) mark for continuous and contactless measurement of HR, RR and motion. No adverse events (AEs) related to the system have been reported during 10 years of monitoring.

For peer review only

For peer review only - http://bmjopen.bmj.com/site/about/guidelines.xhtml

Developed in 2019, the advanced neonatal epidermal (ANNE) system from Sibel, a technology company spun out from the Center of Bio-Integrated Electronics at Northwestern University in the United States, is a system of two time-linked soft and flexible sensors designed to measure and monitor vital signs including HR, RR, oxygen saturation (SpO_2), and skin temperature in neonates. The chest sensor couples to the skin via a hypo-allergenic, biocompatible hydrogel adhesive optimized for reduced peel force upon removal, and the limb unit couples via a latex-free soft fabric wrap adaptable to a range of foot sizes and anatomies. Information from the sensors are wirelessly transmitted to a monitor or mobile device via encrypted Bluetooth™ for real-time streaming from a customized mobile software application as well as onboard memory storage on the sensors themselves. The device has been validated in more than 50 neonates in a neonatal care unit without AEs.

Reference devices

We are employing the Masimo Rad-97™ and the Spengler Tempo Easy Bleu devices as our reference devices for this study. The Masimo Rad-97™ provides continuous physiological monitoring of HR, RR, SpO2, and capnography. The Spengler Tempo Easy Bleu non-contact infrared thermometer predicts core body temperature from the temporal artery temperature.

Study procedures

Following completion of screening for eligibility, a study comprehension checklist, and written informed consent, study staff perform procedures according to the most recently approved version of the protocol (current version 1.1, June 18, 2019 (Appendix 1)). Enrolled neonates are assigned a participant identification number; information is collected on socio-

For peer review only - http://bmjopen.bmj.com/site/about/guidelines.xhtml

demographic characteristics, current clinical status, medical history, medications; and a physical examination is performed.

Prior to initiating the accuracy evaluation of each investigational device, verification of the reference devices, Masimo Rad-97™ and Tempo Easy Bleu, is undertaken at AKU-N to ensure they are robustly functional and to confirm their within subject repeatability and accuracy compared to standard clinical measurements for the relevant parameters of interest. Neonates enrolled during reference device verification continue to receive local standard of care while being observed intermittently for vital signs collection for a minimum of 1 hour using the Masimo Rad-97™ and intermittent measurements with the Tempo Easy Bleu. Observations may include video recordings of the neonate and the Masimo Rad-97™reference device monitor for later review to facilitate manual count observations. The reference device measurements will be compared to manual measurements, clinical monitor observations, and video-assisted observations. Reliability information gathered from the reference devices is utilized to determine specific Go/No Go criteria for each parameter and each investigational device. Further evaluation of each investigational device only proceeds should these criteria be met.

Enrollment in the accuracy evaluation of the investigational devices, EarlySense Insight system and Sibel ANNE system, is initiated at AKU-N to formally assess their accuracy compared to the verified reference device using repeated observations. Enrolled neonates continue to receive local standard of care while having vital signs collected from the reference device as well as one or both of the investigational devices. Placement of the investigational and reference devices is done in a manner so as not to interfere with the neonate’s clinical care. Observations are collected for a minimum of 1 hour and potentially

For peer review only - http://bmjopen.bmj.com/site/about/guidelines.xhtml

For peer review only

for the entire duration of their stay in the hospital. Observations may consist of videotaping and/or taking photos of the neonate during the observation period after obtaining informed consent from the caregiver. During observation, clinical status and any activities are updated and recorded including care activities (e.g., feeding, diaper changes, bathing, kangaroo mother care, etc.), clinical procedures, interventions, therapies, laboratory tests, medications, environmental features and exposures during hospitalization. The device placement, output, and signal quality are also monitored. In addition, the neonates are assessed for any safety issues. Agreement between the investigational and reference devices is determined using *a priori*-defined accuracy thresholds. Thresholds are determined based on the repeated observations performed on the reference device in the verification phase, international standards, and clinical expert consensus opinion. Two *a priori*-determined thresholds are determined: one lower threshold to allow the device developer to optimize the device for retesting, and a second higher threshold to allow the device to move on to the clinical feasibility phase of testing. A maximum of 5 rounds of testing and retesting are permitted for each investigational device. Each round of testing or retesting consists of using a cohort of 20 neonates. Should the lower threshold not be reached for at least one parameter, no further testing of the investigational device is performed. Thus, information collected during the accuracy evaluation along with the *a priori*–determined Go/No Go criteria established during verification of the reference devices define which, if any, of the investigational devices moves forward with additional rounds of testing or into the clinical feasibility phase at PMH.

An investigational device advances to the clinical feasibility phase once the agreement for the measurement parameters of interest exceed the higher accuracy threshold. Enrollment

For peer review only - http://bmjopen.bmj.com/site/about/guidelines.xhtml

in the clinical feasibility phase of the investigational devices occurs at PMH in up to 120 enrolled neonates who receive local standard of care while being monitored with the reference device(s) and one or both of the investigational devices. Observations are collected for a minimum of 1 hour and involve measurement of vital signs via the investigational and reference devices and monitoring for any critical event (i.e., low or high HR, RR, or temperature or oxygen desaturation and apnea). Agreement between repeated observations from the investigational and reference devices as well as diagnostic performance in clinical event detection is evaluated. Additional performance metrics such as alarm rates, alarm delays and uptime\downtime is compared between the investigational and reference devices. Participation in the study does not interfere with or unnecessarily delay the clinical care of the neonates.

Throughout all phases of the research, the investigational devices are not used to inform clinical care. During the clinical feasibility phase, ETNA site study staff and hospital HCPs are blinded to the data collected from the investigational devices to prevent interference with clinical care. The study site investigators are responsible for close safety monitoring of all participating neonates, including assessing for and reporting adverse device effects (e.g., erythema or edema at the investigational or reference device sensor site) and/or serious adverse events (i.e., any adverse device effect resulting in permanent skin damage). Any adverse device effects or serious adverse events will be treated until resolution or stabilization, and may require removal of devices and withdrawal of the neonate from the study if necessary.

Qualitative substudy

For peer review only - http://bmjopen.bmj.com/site/about/guidelines.xhtml

After written informed consent is received from the qualitative study participants, a mixed methods evaluation and data collection through audio-recorded semi-structured in-depth interviews and direct observations are conducted by trained qualitative study staff to assess the feasibility, usability, and acceptability of the investigational devices for monitoring of neonates in an African-setting. All hospital administrators and study HCPs may be involved in this portion of the study. Caregivers with a neonate enrolled in the study may also be asked if they would like to participate in the qualitative portion of the study.

Sample size

A total of up to 500 neonates are enrolled. For the verification of the reference devices at AKU-N, up to 30 neonates are enrolled. Once this initial testing and data collection of the reference devices is complete, for the accuracy evaluation phase at AKU-N, up to 120 neonates per investigational device are enrolled. For the clinical feasibility phase at PMH, up to 120 neonates per investigational device are enrolled. The sample sizes for each phase were selected to maximize the amount of information collected within the confines of the available resources.

For the feasibility, usability, and acceptability assessment, the total sample size includes all hospital administrators and study HCPs willing to participate and provide consent as well as up to 30 caregivers willing to participate and provide consent study at each site.

Data collection and quality assurance

Quantitative study data is collected by clinical study staff using designated source documents as well as electronic or paper-based case report forms. Data is stored and managed by a database developed via Research Electronic Data Capture (REDCap), a secure

For peer review only - http://bmjopen.bmj.com/site/about/guidelines.xhtml

web application. Continuous physiological data and event data are recorded from the investigational and reference devices at least once a second. All electronic data are collected wirelessly or via a wired connection from the investigational and reference devices to a study laptop using custom software applications. Qualitative study data is collected using paper-based forms and audio recordings which are subsequently transcribed for analysis.

Clinical research data, including data collected from the investigational and reference devices, are maintained through a combination of secure electronic data management system and physical files with restricted access to ensure confidentiality. Two distinct study databases are maintained separately: the primary study database and a database with participating neonate's personally identifiable information. To ensure accuracy and completeness, data is routinely reviewed by the sponsor through quality assurance reviews, audits, and evaluation of the study safety and progress. Guideline for Good Clinical Practice (GCP)/ ISO 14155 compliance is followed to ensure accurate, reliable, and consistent data collection.

Data management

Primary data management activities, which include de-identified investigational and reference device data transfer using end-to-end encryption with two-factor authentication, data entry and validation, data cleaning, database quality control, and disaster recovery plans are undertaken at the study site and are overseen by the on-site data manager. Data review and analysis, oversight and preparation of final study database is performed by the sponsor in collaboration with the study site. Data are maintained in databases hosted at the study site. All data management activities are in compliance with International Council on

For peer review only - http://bmjopen.bmj.com/site/about/guidelines.xhtml

Harmonization (ICH) GCP E6, sponsor organization, and institutional requirements for the protection of children and confidentiality of personal and health information.

Outcomes

We hypothesize that the investigational device is accurate and reliable compared to the reference device for each relevant measurement parameter of interest among neonates and is feasible, usable and acceptable for use in neonates in low-resource settings. The primary endpoint and secondary endpoints are detailed in Table 2.

Statistical analyses

Every second of data is automatically graded as optimal, acceptable and unacceptable based on predefined rules for each device and each measurement parameter of interest according to the quality of the data for each measurement parameter of interest. The Masimo Rad-97™ provides a signal quality index that is used to determine data quality for HR and SpO2. A custom algorithm has been produced to determine the capnography signal quality index. Each of the investigational devices also provides a signal quality index. The quality thresholds are determined following the verification phase. All comparisons are performed from observations between two devices (or a single device during the verification phase). At least 10 observations of 60 seconds of optimal quality data in each neonate, at least 5 minutes apart, are randomly selected for each measurement parameter of interest from the full recording. For the clinical feasibility phase, accuracy comparisons use optimal or acceptable data. At least 3 hours of recording to a maximum of 12 hours are used for the performance metrics such as alarm rates, alarm delays and uptime\downtime.

For peer review only

For peer review only - http://bmjopen.bmj.com/site/about/guidelines.xhtml

The repeatability of the reference device parameter estimates initially is assessed with the intraclass correlation coefficient (ICC). Additional training or standardization of procedures is performed to ensure at least good repeatability (ICC >0.7). This is followed by measuring agreement between the repeated reference observations and between the manual, clinical monitor and video-assisted methods and the reference observations using the methods described by Bland and Altman for replicated observations.[6] The agreement is reported as a mean bias with 95% confidence intervals (Cis) and 95% limits of agreement. Graphical representation of the data is assessed with agreement plots, Clark error grids, and Polar plots to identify extreme outliers and significant data trends.

In the accuracy evaluation, the root mean square difference (RMSD) and ICC are calculated for each measurement parameter of interest to compare the multiple repeated observations between the investigational and reference devices. The agreement between each investigational device and reference device(s) is then calculated using the methods described by Bland and Altman for replicated observations. The agreement is reported as a mean bias with 95% CIs and 95% limits of agreement. Graphical representation of the data is be assessed with agreement plots, Clark error grids, and Polar plots to identify extreme outliers, impact on clinical decisions, and significant data trends. An *a priori*–defined accuracy margin for agreement is used as a threshold value to allow for decisions regarding proceeding to additional testing.

In the clinical feasibility phase, agreement between each investigational device and reference device(s) is assessed as in the accuracy evaluation phase. Event detection rates, alarm rates, alarm delays and uptime/ downtime are summarized with means, medians, standard deviations and intra-quartile ranges as appropriate. Summaries of sensitivity,

For peer review only - http://bmjopen.bmj.com/site/about/guidelines.xhtml

specificity, positive predictive values and negative predictive values comparing each measurement parameter of interest in the investigational device(s) to the reference device(s) are produced. Comparisons of binary events are assessed using Cohen's weighted Kappa and McNemar's test. The non-inferiority of alarm rates, alarm delays and uptime/ downtime are evaluated based on pre-specified thresholds.

Qualitative data are collected through in-depth interviews and/or semi-structured questionnaires and analyzed to assess feasibility, usability, and acceptability of the investigational devices among hospital administrators and HCPs, and acceptability among caregivers of enrolled neonates. The qualitative data is in narrative format and the results are descriptive. The questionnaires are coded and analyzed using a codebook with identified themes, including feasibility of using each investigational device, barriers and facilitators to use, and perceived value. Qualitative data analysis software is used to organize, code, and analyze the qualitative data in an iterative process. The study team starts by identifying an initial set of codes and themes based on the categories from the interview guides. During the coding process, attention is paid to identifying emergent issues and themes that are added to the codebook and included in the analysis. Responses from the interviews are coded and discrepancies discussed and resolved for the final analysis and theme identification.

ETHICS AND DISSEMINATION

Ethical approvals and consent

For peer review only

For peer review only - http://bmjopen.bmj.com/site/about/guidelines.xhtml

363 The study is conducted in accordance with the ICH GCP and the Declaration of Helsinki 2008. 364 The protocol and other relevant study documents study were approved by the Western 365 Institutional Review Board (Puyallup, Washington, United States of America), and the Aga 366 Khan University Nairobi Research Ethics Committee (Nairobi, Kenya). Written informed 367 consent is obtained in the local language by trained study staff from all eligible neonate's 368 caregivers and for the qualitative substudy, from participating hospital administrators, HCPs, 369 and caregivers prior to enrollment.

370 **Possible risks**

371 Caregivers may feel compelled to enroll in the study in order to receive care for their 372 neonate within a research setting, which may be perceived as of a higher quality than the 373 standard of care. In order to minimize the risk of coercion, during the informed consent 374 process, study staff emphasize that the neonate will receive the required medical care 375 whether enrolled in the study or not. Other potential risks to study participation may 376 include those associated with the placement and attachment of the investigational and 377 reference devices, and delayed medical management. Study staff are trained in the 378 appropriate placement of investigational and reference devices' sensors to minimize 379 discomfort to the neonates as well as to avoid interference with any assessment, treatment, 380 or intervention necessary for clinical care. There is a potential risk of skin irritation with the 381 ANNE sensor system and neonates will be closely monitored and treated for any AEs. Study 382 staff are also trained in integrating study procedures with clinical care and to always 383 prioritize clinical care above study procedures. Extreme care is taken to ensure that no 384 necessary treatment is delayed to accommodate study procedures.

385 **Dissemination**

For peer review only

For peer review only - http://bmjopen.bmj.com/site/about/guidelines.xhtml

386 We plan to disseminate study results in peer-reviewed journals and international
387 conferences, targeting those involved in the clinical care of neonates in low-resource
388 settings as well as those who develop and advise on policies and guidelines in those settings.
389 The trial is registered with ClinicalTrials.gov (registration number NCT03920761).

390 **Efforts towards rigorous protocol**

391 Dedicated study staff trained in GCP, operation, use and maintenance of the investigational
392 and reference devices, and study-specific procedures follow neonates enrolled in the trial to
393 assure the protocol and standard operating procedures are followed and data are accurately
394 collected. Standardized training, supervision, and oversight are undertaken to ensure
395 quality, consistency, and harmonized trial procedures and implementation. Regular
396 monitoring is provided by Save the Children to assess compliance with human subjects and
397 other research regulations and guidelines, adherence to the study protocol and procedures,
398 and quality and accuracy of data collected.

399 **Limitations and bias**

400 Limitations to this study and potential sources of bias include the sampling strategy, the
401 uncertainty inherent in the measurements from the reference devices, the limited
402 standardization of time of day of recording, and the inability to control the conditions and
403 standardize the context. Because there is a large variation in the various ages, weights, sizes,
404 disease states, clinical presentations, interventions received, and conditions of the
405 participating neonates, it is not possible to control for all these variables. Likewise, the
406 environment cannot be controlled, does not allow for complete standardization, and may
407 introduce additional sources of bias.

For peer review only

For peer review only - http://bmjopen.bmj.com/site/about/guidelines.xhtml

DECLARATIONS

Authors' contributions ASG, EN, WM and MA designed the study and wrote the protocol. RO, MW, GZ, and SX reviewed and provided critical input to the study design and protocol. ASG wrote the first draft of the manuscript, and EN and MA provided additional input. The authors worked collaboratively and made the decision to submit the final manuscript for publication.

Acknowledgments We would like to thank Dustin Dunsmuir who wrote the IAP logger application used to collect the high resolution data from the reference device.

Funding This work is supported by grants from the Bill & Melinda Gates Foundation (OPP1203136) and the Save the Children Innovation Council. The authors had final responsibility for the decision to submit this manuscript for publication.

Competing interests RK is employed by EarlySense Ltd. and SX is employed by Sibel Inc. All other authors declare that they have no competing interests.

Ethics approval and consent to participate The study was approved by the Western Institutional Review Board (Puyallup, Washington, United States of America) and the Aga Khan University Nairobi Research Ethics Committee (Nairobi, Kenya). Written informed consent is obtained by trained study staff from all eligible children's caregivers prior to enrollment.

Consent for publication Not applicable.

Patient and public involvement Patients and the public were not involved in the design of, recruitment to, or the conduct of the study.

Availability of data and materials Data will be made available on an open access platform

For peer review only

For peer review only - http://bmjopen.bmj.com/site/about/guidelines.xhtml

after the publication of the main manuscripts. Processes will be developed to facilitate data sharing for scientific utilization in a collaborative manner.

For peer review only

For peer review only - http://bmjopen.bmj.com/site/about/guidelines.xhtml

REFERENCES

1. United Nations Inter-agency Group for Child Mortality Estimation (UN IGME), 'Levels & Trends in Child Mortality: Report 2018, Estimates developed by the United Nations Inter-agency Group for Child Mortality Estimation' United Nations Children's Fund. New York, 2018.

2. Sustainable Development Goals Knowledge Platform. Division for Sustainable Development Goals.[website]. New York, NY: United Nations Headquarters. Date Accessed: September 22, 2019. https://sustainabledevelopment.un.org/sdg3.

3. Hug L, Alexander M, You D, Alkema L, Estimation UNI-aGfCM. National, regional, and global levels and trends in neonatal mortality between 1990 and 2017, with scenario-based projections to 2030: a systematic analysis. *Lancet Glob Health* 2019; **7**(6): e710-e20.

4. Zhu Z, Liu T, Li G, Li T, Inoue Y. Wearable sensor systems for infants. *Sensors (Basel)* 2015; **15**(2): 3721-49.

5. Sahni R. Continuous noninvasive monitoring in the neonatal ICU. *Curr Opin Pediatr* 2017; **29**(2): 141-8.

6. Bland JM, Altman DG. Measuring agreement in method comparison studies. *Stat Methods Med Res* 1999; **8**(2): 135-60.

For peer review only - http://bmjopen.bmj.com/site/about/guidelines.xhtml

TABLES AND FIGURE LEGENDS

Table 1. Eligibility criteria

**Eligibility criteria**	
Inclusion criteria	• Male or female neonate, corrected age of ≥ 28 days.<br>• Willingness and ability of neonate’s caregiver to provide informed consent and to be available for follow-up for the planned duration of the study.
Exclusion criteria	• Receiving mechanical ventilation or continuous positive airway pressure.<br>• Skin abnormalities in the nasopharynx and/or oropharynx.<br>• Contraindication to application of skin sensors.<br>• Known arrhythmia.<br>• Presence of a congenital abnormality requiring major surgical intervention.<br>• Any medical or psychosocial condition or circumstance that, in the opinion of the investigators, would interfere with the conduct of the study or for which study participation might jeopardize the neonate’s health.

For peer review only

Table 2. Study endpoints

**Primary endpoints**
• Agreement of the relevant measurement parameters of interest between the investigational device and the reference device at each observation.
• Agreement of clinical event detection between the investigational device and the reference device at each observation.
• Feasibility, usability and acceptability of the investigational device among hospital administrators and healthcare providers.
• Acceptability of the investigational device among caregivers.
**Secondary endpoints**
• Diagnostic performance of the investigational device to appropriately identify the following critical events:<br>○ Low heart rate<br>○ High heart rate<br>○ Low respiratory rate<br>○ High respiratory rate<br>○ Oxygen desaturation<br>○ Apnea<br>○ Low temperature<br>○ High temperature
• Downtime duration of the investigational device.
• Alarm rate (events/hour and ratio of false positives to missed critical events of the investigational device's alarms compared to the reference device's alarms.

For peer review only

For peer review only - http://bmjopen.bmj.com/site/about/guidelines.xhtml

- Response time of the investigational device’s alarms compared to the reference device’s alarms for critical events.
- Proportion of neonates with adverse device effects and serious adverse events resulting in skin damage.

453

For peer review only

For peer review only - http://bmjopen.bmj.com/site/about/guidelines.xhtml

APPENDICES

Appendix I: Protocol, version 1.1, June 18, 2019

Appendix II: Schedule of study procedures and evaluations

Activity	Screening	Enrollment	Observation	Discharge
Eligibility assessment	X			
Informed consent and comprehension checklist	X			
Assign participant ID	X			
Demographics	X	X		
Medical history		X	X	X
Maternal pregnancy history		X		
Medications use		X		
Placement of reference and/or investigational device(s)		X	X	
Collection of vital signs		X	X	X

For peer review only

For peer review only - http://bmjopen.bmj.com/site/about/guidelines.xhtml

Video tape recording and/or photographs			X	
Track clinical care and non-study activities			X	X
Safety assessment			X	
End of study questions				X
Removal of investigational and/or reference device(s)				X

For peer review only

For peer review only - http://bmjopen.bmj.com/site/about/guidelines.xhtml

Evaluation of Technologies for Neonates in Africa (ETNA) project

Full title: Evaluation of technologies for neonates in Africa

A Study of the Save the Children Technology Accelerator Unit
Sponsored by:
Save the Children Federation, Inc., United States

Version 1.1
18 June 2019

Local Principal Investigator: William Macharia, MBChB,MMed, MSc
Principal Investigators: Amy Ginsburg, MD, MPH and J. Mark Ansermino, MBBCh

**Investigators**	**Investigators**
**Co-Investigator:** Zvika Shinar<br>**Co-Investigator:** Shuai Xu, MD	**Co-investigator:** Roseline Ochieng, MBChB, MMed<br>**Co-investigator:** Mary Waiyego, MBChB,MMed

Confidentiality Statement

This document is confidential and is to be distributed for review only to investigators, potential investigators, consultants, ETNA study staff, applicable regulatory authorities, and applicable independent ethics committees or institutional review boards. The contents of this document shall not be disclosed to others without written authorization from Save the Children (or others, as applicable), unless it is necessary to obtain informed consent from potential ETNA study participants.

For peer review only

For peer review only - http://bmjopen.bmj.com/site/about/guidelines.xhtml

Evaluation of Technologies for Neonates in Africa (ETNA) project

TABLE OF CONTENTS

TABLE OF CONTENTS 2
ABBREVIATIONS AND ACRONYMS 5
PROTOCOL TEAM 7
PARTICIPATING INSTITUTIONS AND CONTACT INFORMATION 8
PROTOCOL OUTLINE 9
1 INTRODUCTION 10
1.1 Background 10
1.2 Rationale 11
1.3 Investigational Devices 11
1.3.1 EarlySense Insight System, EarlySense Ltd 11
1.3.2 Advanced Neonatal Epidermal System, Sonica 12
1.4 Reference Devices 12
2 STUDY HYPOTHESES, OBJECTIVES AND ENDPOINTS 12
2.1 Study Hypotheses 12
2.2 Study Objectives 12
2.2.1 Primary Objectives 12
2.2.2 Secondary Objectives 13
2.3 Study Endpoints 13
2.3.1 Primary Endpoints 13
2.3.2 Secondary Endpoints 13
3 METHODOLOGY 14
3.1 Study Design 14
3.2 Study Sites 15
3.2.1 Aga Khan University – Nairobi 15
3.2.2 Pumwani Maternity Hospital 15
3.3 Study Population 16
3.3.1 Study Population Overview 16
3.3.2 Participant Eligibility 16
3.3.3 Sample Size 16
3.4 Study Period 17
4 STUDY PROCEDURES 17
4.1 Recruitment 17
4.2 Screening 17
4.3 Informed Consent Process 17
4.4 Enrollment 18
4.5 Observations 18
4.6 Interrupted or Missed Observations 19
4.7 Withdrawal and Early Termination 19

For peer review only

4.8 Study Termination 20
5 STUDY DEVICES 20
5.1 Descriptions 20
5.2 Method for Assigning Participants 20
5.3 Placement of the Devices 20
5.4 Blinding of Study 21
5.5 Packaging 21
5.6 Receiving, Storage, Dispensing and Return 21
6 DATA COLLECTION 21
6.1 Case Report Forms 21
6.2 Questionnaires 22
6.3 Source Documents 22
6.4 Device Data Collection 22
6.5 Data Management 22
6.6 Data Access 23
6.7 Data Storage 23
7 SAFETY ASSESSMENT AND MONITORING 23
7.1 Safety Monitoring 23
7.2 Adverse Device Effect 23
7.3 Serious Adverse Events 24
7.4 Adverse Event Relationship to Devices 24
7.5 Grading Severity of Events 24
7.6 Safety Reporting 24
8 TRAINING REQUIREMENTS, MONITORING AND REPORTING 25
8.1 Training Requirements 25
8.2 Monitoring 25
8.3 Study Discontinuation 25
9 STATISTICAL DESIGN AND ANALYSIS 25
9.1 Overview and General Design 25
9.2 Analytical Methodology 26
9.3 Sample Size Estimation 27
10 RESULT PRESENTATION 28
10.1 Dissemination of Results 28
11 ETHICAL CONSIDERATIONS AND CONSENT 28
11.1 Principles for Clinical Research 28
11.2 Institutional Review Boards and Independent Ethics Committees 28
11.3 Informed Consent Documentation 29
11.4 Risks and Benefits 29
11.4.1 Risks to Participants 29
11.4.2 Protection against Risks 30
11.4.3 Benefits to Participants 30
11.5 Participant Confidentiality 30
12 POSSIBLE CONSTRAINTS 30
PROTOCOL REFERENCES 32

For peer review only - http://bmjopen.bmj.com/site/about/guidelines.xhtml

APPENDICES 33
Appendix I: Schedule of Study Procedures and Evaluations 33

For peer review only

For peer review only - http://bmjopen.bmj.com/site/about/guidelines.xhtml

Evaluation of Technologies for Neonates in Africa (ETNA) project

ABBREVIATIONS AND ACRONYMS

ADE	adverse device effect
AE	adverse event
AKU-N	Aga Khan University, Nairobi
ANNE	advanced neonatal epidermal system
CE	Conformité Europééne
CI	confidence interval
CRF	case report form
CPAP	continuous positive airway pressure
EMC	electromagnetic compatibility
ETNA	Evaluation of Technologies for Neonates in Africa
FDA	United States Federal Drug Administration
GCP	good clinical practice
HCP	healthcare provider
HR	heart rate
ICC	intraclass correlation coefficient
ICF	informed consent form
ID	identification
IDI	in-depth interview
IEC	International Electrotechnical Commission
IRB	institutional review board
ISO	International Organization for Standardization
LAR	legally authorized representative
LRS	low-resource settings
nHDU	neonatal high dependency unit

For peer review only - http://bmjopen.bmj.com/site/about/guidelines.xhtml

NICU	neonatal intensive care unit
NSR	non-significant risk
PI	principal investigator
PMH	Pumwani Maternity Hospital
QC	quality control
RMSD	root mean square difference
RR	respiratory rate
SAE	serious adverse event
SCUS	Save the Children Federation Inc., United States
SDG	Sustainable Development Goal
SOP	standard operating procedure(s)
SpO2	oxygen saturation
WIRB	Western Institutional Review Board

For peer review only

For peer review only - http://bmjopen.bmj.com/site/about/guidelines.xhtml

Evaluation of Technologies for Neonates in Africa (ETNA) project

PROTOCOL TEAM

Principal Investigator (PI) and Medical Officer:
Amy Ginsburg, MD, MPH

Senior Advisor, International Programs ______________________ PI signature Date
Save the Children Federation, Inc., United States

Co-PIs:
William Macharia, MBChB,MMed, MSc
Aga Khan University, Nairobi, Kenya

J. Mark Ansermino, MBBCh
University of British Columbia, Canada

Co-Investigators:
Roseline Ochieng, MBChB, MMed
Aga Khan University, Nairobi, Kenya

Mary Waiyego, MBChB, MMed
Aga Khan University, Nairobi, Kenya

Zvika Shinar
EarlySense

Shuai Xu, MD
Sonica

Biostatistician:
Guohai Zhou, PhD

Research Manager:
Evangelyn Nkwopara, MS

For peer review only

PARTICIPATING INSTITUTIONS AND CONTACT INFORMATION

**Study Oversight/ Management:**	Save the Children Federation, Inc., United States (SCUS)<br>501 Kings Highway East, Suite 400<br>Fairfield, CT 06825
**Medical Officer:**	Amy Ginsburg, MD, MPH<br>Save the Children Federation, Inc. (SC)<br>501 Kings Highway East, Suite 400<br>Fairfield, CT 06825<br>+1-206-258-1827<br>aginsburg@savechildren.org
**Study Sites:**	Aga Khan University Hospital, Nairobi (AKU-N)<br>3rd Parklands Avenue, Limuru Road, Nairobi, Kenya<br><br>Pumwani Maternity Hospital (PMH)<br>Muratina Street Nairobi, Kenya
**Study Operations:**	Aga Khan University, Nairobi, Kenya
**Local Collaborators:**	Aga Khan University, Nairobi, Kenya<br>Pumwani Maternity Hospital, Nairobi, Kenya
**Statistical Support:**	Guohai Zhou, PhD
**Technical Support:**	**EarlySense**<br>US Office:<br>800 West Cummings Park<br>Suite 6400, Woburn, MA 01801 United States<br><br>International Office:<br>Derech Ze'ev Jabotinsky 7,<br>Ramat Gan, 5252007 Israel<br><br>**Sonica**<br>1900 Greenwood Street, Suite 2,<br>Evanston, Illinois 60201 United States
**Funding Agency:**	The Bill & Melinda Gates Foundation<br>500 Fifth Avenue North<br>Seattle, WA 98109

For peer review only

PROTOCOL OUTLINE

<table>
<tr><td>Title:</td><td>Evaluation of technologies for neonates in Africa</td></tr>
<tr><td>Study Oversight and Management:</td><td>Save the Children Federation Inc., United States</td></tr>
<tr><td>Collaborating Organizations:</td><td>Save the Children Federation Inc., United States (SCUS)
Aga Khan University – Nairobi (AKU-N)
Pumwani Maternity Hospital (PMH)</td></tr>
<tr><td>Funding Sources:</td><td>Bill & Melinda Gates Foundation</td></tr>
<tr><td>Rationale:</td><td>Low-cost, operator-independent, efficient, non-invasive multiparameter continuous physiological monitoring devices are necessary for use and need to be evaluated in neonates in a low resource settings.</td></tr>
<tr><td>Population:</td><td>Neonates at AKU-N and PMH</td></tr>
<tr><td>Schema:</td><td>Up to 500 children total will be enrolled in the following manner:
<table>
<tr><th>Study phase</th><th>N</th><th>EarlySense</th><th>Sonica</th></tr>
<tr><td>Phase I: verification</td><td>20</td><td>N/A</td><td>N/A</td></tr>
<tr><td>Phase I: accuracy evaluation</td><td>240*</td><td>120</td><td>120</td></tr>
<tr><td>Phase II: clinical feasibility</td><td>240</td><td>120</td><td>120</td></tr>
</table>
* 5 rounds of testing and retesting per investigational device, 20 neonates per round</td></tr>
<tr><td>Objectives:</td><td>Primary:
1. To assess agreement between repeat observations by the investigational device and the reference device(s) for each relevant measurement parameter of interest based on a priori-determined non-inferiority margins among neonates.
2. To compare clinical event detection performance between the investigational device and the reference device(s).
3. To determine whether the investigational device is feasible, usable and acceptable among healthcare providers, hospital administrators, and caregivers of neonates.

Secondary:
1. To assess diagnostic performance for each relevant measurement parameter of interest based on sensitivity, specificity, positive predictive value, and negative predictive value compared to the reference device(s).
2. To determine the downtime performance of the investigational device.
3. To determine the alarm rate (events/hour) and the number of true/false alarms of the investigational device compared to the reference device(s).
4. To determine the delay time between the investigational device and the reference device(s) in true events.
5. To determine the number of adverse device effects and serious adverse events resulting in skin damage during use of the investigational device.</td></tr>
</table>

For peer review only - http://bmjopen.bmj.com/site/about/guidelines.xhtml

Endpoints:	Primary<br>1. Agreement of the relevant measurement parameters of interest between the investigational device and the reference device(s) at each observation.<br>2. Agreement of clinical event detection between the investigational device and the reference device(s) at each observation.<br>3. Feasibility, usability and acceptability of the investigational device among healthcare providers and hospital administrators.<br>4. Acceptability of the investigational device among caregivers.<br><br>Secondary<br>1. Diagnostic performance of the investigational device to appropriately identify the following critical events:<br>a. Low heart rate<br>b. High heart rate<br>c. Low respiratory rate<br>d. High respiratory rate<br>e. Oxygen desaturation<br>f. Apnea<br>g. Low temperature<br>h. High temperature<br>2. Downtime duration of the investigational device.<br>3. Alarm rate (events/hour) and ratio of false positives to missed critical events of the investigational device's alarms compared to the reference device(s)' alarms.<br>4. Response time of the investigational device's alarms compared to the reference device(s)' alarms for critical events.<br>5. Proportion of neonates with adverse device effects and serious adverse events resulting in skin damage.
Timeline:	Total project anticipated to take 18 months to complete.

1 INTRODUCTION

1.1 Background

In 2017 globally, 47% of all deaths in children under five years of age occurred within the first 28 days of life, which translates to a neonatal mortality rate of 18 deaths per 1000 live births.[1] Sub-Saharan Africa bears the greatest burden of neonatal mortality with an estimated 1 million newborn deaths in 2017.[1] Further efforts, especially in African countries, are needed to push progress towards achieving the Sustainable Development Goal (SDG) 3 target of reducing global neonatal mortality to 12 deaths per 1000 live births by 2030.[2] Innovations in neonatal care, particularly technologies that allow for early detection and intervention of major morbidities, hold great promise in helping to reduce current neonatal mortality rates.

For peer review only - http://bmjopen.bmj.com/site/about/guidelines.xhtml
For peer review only

Multiparameter continuous physiological monitoring devices could be instrumental in directing care provided for a neonate through automatic interpretations of vital signs that help identify critical events and determine if treatment is sufficient or insufficient, ultimately improving newborn outcomes.[3-4] These devices would be most useful in low-resource settings (LRS) in sub-Saharan African were the need for such technologies is greatest. While such devices currently exist and are standard of care in high-resource settings, they are expensive and require specialized training to operate, making them unsuitable for application in LRS. To address these barriers, it is necessary to explore how these technologies can be adapted and/or optimized for use in LRS. Ideally the devices should be low cost, operator-independent, and highly efficient in diagnostic performance and operator workload. This requires development of a robust testing platform that appropriately mimics conditions common in an African newborn unit or neonatal intensive care unit (NICU) that would allow these type of technologies to be evaluated for performance and feasibility.

To this end, the Evaluation of Technologies for Neonates in Africa (ETNA) project was conceived with the goal of advancing and managing development, as well as evaluation, of select devices for use in neonates. By establishing a testing platform in an African site, and working collaboratively with partners with expertise in device development and evaluation and neonatal and child health, the project seeks to boost development and optimization of promising newborn care devices that could be applied in LRS in Africa.

1.2 Rationale

To further reduce neonatal mortality rate in LRS in Africa, research is needed to develop and optimize innovations in newborn care, specifically technologies that are low cost, operator-independent, and highly efficient. The purpose of the ETNA project and this initial study is to produce information and data regarding the performance of two existing multiparameter continuous physiological monitoring devices developed by device developers, EarlySense and Sonica. The clinical trial described in this protocol is intended to provide evidence to establish whether these investigational devices can reliably and accurately measure vital signs in neonates (when compared to verified reference devices) and to assess the feasibility, usability and acceptability of these devices for use in neonates in a LRS in Africa.

1.3 Investigational Devices

1.3.1 EarlySense Insight System, EarlySense Ltd.

The EarlySense Insight system is a contact-free monitoring system, that measures and records a patient's vital signs and motion parameters. The system was developed by Israeli-based EarlySense. The system is comprised of a small piezoelectric sensor pad that can be placed under the patient's mattress to monitor heart rate (HR), respiratory rate (RR), patient motion, and sleep status. There is no physical contact between the sensor and the patient. Information from the sensor pad, in combination with EarlySense's artificial intelligence analytics, is transmitted to a monitor or mobile application to provide alert indications and vital sign trends to healthcare providers (HCPs) so that they can monitor changes in a patient's condition. These alert indications, together with EarlySense artificial intelligence capabilities are able to detect sepsis, respiratory depression, sleep apnea, and arrhythmias, among other conditions. This device is currently in use in hospitals, rehabilitative centers, and nursing homes to measure vital signs in children and adults above 10 kg but will be modified for use in neonates as part of

For peer review only - http://bmjopen.bmj.com/site/about/guidelines.xhtml

this study. The adult device received regulatory approval from the United States Federal Drug Administration (FDA) and has a Conformité Européene (CE) mark for continuous and contactless measurement of HR, RR and motion.

The EarlySense system has been subject to full verification and validation tests that include:

1. Risk analysis
2. Software verification and validation
3. Electromagnetic compatibility (EMC) and safety testing
4. Full load bench testing
5. Non clinical testing: EarlySense Insight performance verification

The electrical safety and electromagnetic compatibility of the EarlySense system (InSight and sensor) were tested by external laboratories to demonstrate that the system is compatible with the requirements of the International Electrotechnical Commission (IEC) 60601-1 and IEC 60601-1-2 standards. Risk analysis activities were performed in compliance with the requirements of International Organization for Standardization (ISO) 14971 “Application of risk management to medical devices.” It was concluded that the potential risks of the EarlySense InSight system are minimal and acceptable. It is considered as a non-significant risk device (NSR). Hundreds of these systems have been installed in healthcare institutions (hospitals, long-term care and skilled nursing facilities) and thousands of patients have been safely monitored. No adverse events (AEs) related to the system have been reported during all years of monitoring.

1.3.2 Advanced Neonatal Epidermal System, Sonica

Sonica, a small technology group based out of Northwestern University in Evanston, Illinois, United States, has developed a system of neonatal non-invasive adhesive sensors that allow wireless, advanced monitoring for neonatal intensive care. This advanced neonatal epidermal (ANNE) system consists of one sensor which contains Bluetooth technology with a built-in battery and a second sensor that is battery-free and ultra-thin. The sensors can be attached directly on the patient’s body and are capable of continuously measuring and recording HR, RR, oxygen saturation (SpO2), and skin temperature. Information from the sensors are wirelessly transmitted to a monitor or mobile device and the monitoring is supported by customized software.

The Sonica ANNE system utilizes existing off-the-shelf, hypo-allergenic adhesives manufactured by Cardinal Health.[5] This hydrogel material has low allergenicity and low peel force. It is used worldwide as an adhesive for neonatal applications. The device itself is current-isolated with no energy or current being delivered from the device to the neonate. It has been validated in more than 50 neonates cared for in the neonatal care unit without a single AE.

1.4 Reference Devices

The Rad-97™ device, developed by Masimo, provides continuous physiological monitoring in a compact, portable, device. The device is capable of monitoring HR, RR, SpO2, and capnography in adult, pediatric, and neonatal patients. Patient information can be transmitted from the device via Ethernet or USB port to wired infrastructures such as traditional nurse call systems or wirelessly via Wi-Fi or Bluetooth technology. This device is similar to devices such

For peer review only - http://bmjopen.bmj.com/site/about/guidelines.xhtml

as blood pressure monitors or cardiopulmonary monitors (monitors HR and RR) that wirelessly communicate, via Wi-Fi or Bluetooth technology, information regarding a newborn's vital signs to a hospital's patient monitoring system within a newborn care unit and NICU.

Temperature will be measured using the Tempo Easy Bleu by Spengler. This non-contact infrared thermometer predicts core body temperature from the temporal artery temperature at a distance of 3-5 cm from the forehead as well as surface or skin temperature.

2 STUDY HYPOTHESES, OBJECTIVES AND ENDPOINTS

2.1 Study Hypotheses

- The investigational device is non-inferior to the reference device(s) for each relevant measurement parameter of interest among neonates.
- The investigational device is feasible, usable and acceptable for use in neonates in a LRS.

2.2 Study Objectives

2.2.1 Primary Objectives

- To assess agreement between repeat observations by the investigational device and the reference device(s) for each relevant measurement parameter of interest based on *a priori*-determined non-inferiority margins among neonates.
- To compare clinical event detection performance between the investigational device and the reference device(s).
- To determine whether the investigational device is feasible, usable and acceptable among HCPs, hospital administrators and caregivers of neonates.

2.2.2 Secondary Objectives

- To assess diagnostic performance for each relevant measurement parameter of interest based on sensitivity, specificity, positive predictive value, and negative predictive value compared to the reference device(s).
- To determine the downtime performance of the investigational device.
- To determine the alarm rate (events/hour) and the number of true/false alarms of the investigational device compared to the reference device(s).
- To determine the delay time between the investigational device and the reference device(s) in true events.
- To determine the number of adverse device effects (ADEs) and serious adverse events (SAEs) resulting in skin damage during use of the investigational device.

For peer review only - http://bmjopen.bmj.com/site/about/guidelines.xhtml

2.3 Study Endpoints

2.3.1 Primary Endpoints

- Agreement of the relevant measurement parameters of interest between the investigational device and the reference device(s) at each observation.
- Agreement of clinical event detection between the investigational device and the reference device(s) at each observation.
- Feasibility, usability and acceptability of the investigational device among HCPs and hospital administrators.
- Acceptability of the investigational device among caregivers.

2.3.2 Secondary Endpoints

- Diagnostic performance of the investigational device to appropriately identify the following critical events:
  a. Low HR
  b. High HR
  c. Low RR
  d. High RR
  e. Oxygen desaturation (SpO2)
  f. Apnea
  g. Low temperature
  h. High temperature
- Downtime duration of the investigational device.
- Alarm rate (events/hour and ratio of false positives to missed critical events of the investigational device's alarms compared to the reference device(s)' alarms.
- Response time of the investigational device's alarms compared to the reference device(s)' alarms for critical events.
- Proportion of neonates with ADEs and SAEs resulting in skin damage.

3 METHODOLOGY

3.1 Study Design

This is a diagnostic accuracy evaluation and clinical feasibility study of investigational devices (EarlySense and ANNE systems) in a neonatal high dependency unit (nHDU) in a private teaching hospital and a government maternity hospital in Nairobi, Kenya. Neonates who are admitted for routine observation and care will be enrolled.

The project consists of two phases: 1. a verification and accuracy evaluation phase and 2. a clinical feasibility phase.

Phase I: The first phase of the study will be conducted at Aga Khan University – Nairobi (AKU-N) nHDU. Prior to initiating the accuracy evaluation of each investigational device, verification of the reference devices (Masimo Rad-97™ and Tempo Easy Bleu) will be undertaken to ensure they are robustly functional and to confirm their within subject repeatability. Conducting

For peer review only

this verification will be critical to informing subsequent activities and Go/No Go criteria in the study. Neonates enrolled during reference device verification will continue to receive local standard of care while being observed intermittently for vital signs collection for a minimum of 1 hour using the Masimo Rad-97™ and intermittent measurements with the Tempo Easy Bleu. Observations will include video recordings of the neonate and the Masimo reference device monitor for later review to facilitate manual count observations in order to determine the repeatability of the reference device. Reliability information gathered from the reference devices will be utilized to determine the specific Go/No Go criteria for each investigational device. Further evaluation of each investigational device will only proceed should these criteria be met.

Enrollment in the accuracy evaluation of the investigational devices, EarlySense system and ANNE system, will be initiated to formally assess their accuracy compared to the verified reference device(s). Enrolled neonates will continue to receive local standard of care while having vital signs collected from the reference device(s) as well as one or both of the investigational devices. Placement of the reference and investigational devices will be done in a manner that will not interfere with the neonate's clinical care. Observations will be collected for a minimum of 1 hour and will consist of videotaping and/or taking photos of the neonate during the observation period after obtaining informed consent from the caregiver. Information collected during the accuracy evaluation along with the *a priori*–determined Go/No Go criteria established during verification of the reference devices will define which, if any, of the investigational devices will move forward with additional rounds of testing or into the clinical feasibility phase at Pumwani Maternity Hospital (PMH).

Phase II: Enrollment in the clinical feasibility phase will occur in PMH's newborn unit. Similar to the verification and accuracy evaluation phase at AKU-N, enrolled neonates will receive local standard of care while being monitored with the reference device(s) as well as one or both of the investigational devices. Observations will be collected for a minimum of 1 hour and involve measurement of vital signs via the reference and investigational devices and monitoring for any critical event (i.e., low or high heart rate, respiratory rate or temperature or oxygen desaturation and apnea). Participation in the study will not interfere with or unnecessarily delay the clinical care of the neonates. However, the investigational devices will not be used to inform clinical care. During the clinical feasibility phase, ETNA site study staff and PMH HCPs will be blinded to the data collected from the investigational devices to prevent interference with clinical care.

Qualitative data collection will also be done to assess the feasibility, usability, and acceptability of the investigational devices) for monitoring of neonates in an African-setting. In-depth interviews (IDIs) and direct observations will take place at each site to assess feasibility, usability, and acceptability. Each IDI will be audio recorded. All ETNA study HCPs and hospital administrators may be involved in this portion of the study. All caregivers with a neonate enrolled in the study may also be asked if they would like to participate in the qualitative portion of the study.

3.2 Study Sites

3.2.1 Aga Khan University – Nairobi

Aga Khan University – Nairobi (AKU-N) is a private, not-for-profit university teaching hospital that receives referrals from both within and outside Kenya in the Eastern and Central African

For peer review only - http://bmjopen.bmj.com/site/about/guidelines.xhtml

region. AKU-N is Joint Commission International accredited and moving to Academic Medical Center standards in the next review. AKU-N sees approximately 650,000 patients in 45 outpatient clinics, 120,000 patients in the emergency department, performs approximately 12,000 surgeries, and delivers 4500 babies per year. The AKU- N NICU is the first in the East African region and has a 4-bed capacity but can be expanded to 8 beds when necessary. Neonates weighing <1000 grams are cared for in the NICU while sick neonates weighing >1000 grams are placed in the nHDU. Neonates in the nHDU are not critically ill but still require close monitoring, typically with continuous physiological monitoring. The AKU-N nHDU admits 5-10 neonates per week and the median length of stay is about 7 days. The nHDU has an 8 bed capacity and the nurse to neonate ratio is approximately 1:3. AKU-N also houses the busiest private hospital maternity unit in Nairobi where neonates may also be observed and monitored in the post-natal ward. Staffing at AKU-N includes 2 full-time faculty neonatologists, 3 private neonatologists, 2 full-time pediatric surgeons, a critical care pediatrician, a pediatric pulmonologist supported by a senior resident and 4 junior residents. NICU-trained and non-trained nurses work in mixed shifts for expertise support. Primary reasons for admissions to the nHDU include: prematurity; asphyxia; respiratory distress syndrome; transient tachypnea; sepsis; jaundice; hypoglycemia; complex congenital heart disease; meconium aspiration syndrome; low birth weight; congenital anomalies; congenital diaphragmatic hernia; and persistent pulmonary hypertension.

3.2.2 Pumwani Maternity Hospital

Founded in 1926 and currently under management of the Nairobi County government, Pumwani Maternity Hospital (PMH) is the largest referral maternity hospital in sub-Saharan Africa (reportedly the third busiest maternity hospital on the continent) and serves a population of 5 million in Nairobi and the surrounding area. PMH has 354 obstetric beds, 144 baby cots, and 50-100 daily deliveries (10-15 Caesarean sections). PMH is a training center for kangaroo mother care which is widely practiced at the facility. Neonates are managed in shared incubators with oxygen in the same room as their mothers, and mothers are also enlisted to perform nursing duties. PMH has no NICU or nHDU, no neonatal facilities for cardiorespiratory support, and no functional microbiology laboratory. There are on average 75 babies in the newborn unit daily with an average length of stay of 5-7 days. Sick neonates include those with hypoxia and suspected sepsis. PMH is run by a medical superintendent who is also a pediatrician and is staffed by 23 nurses, with 3 nurses working per shift. The nurse per neonate ratio is 1:25 and 6-7 out of the 25 neonates are sick.

3.3 Study Population

3.3.1 Study Population Overview

Enrolled neonates at each site will be representative of the ethnic demographics in Nairobi, Kenya. Both female and male neonates will be enrolled for a total participant population of up to 500 neonates.

3.3.2 Participant Eligibility

The study will enroll neonates admitted for routine observation and care at AKU-N or PMH.

<u>Inclusion Criteria</u>

For peer review only - http://bmjopen.bmj.com/site/about/guidelines.xhtml

1. Male or female neonate, corrected age of ≤ 28 days.
2. Willingness and ability of neonate's caregiver to provide informed consent and to be available for follow-up for the planned duration of the study.

Exclusion Criteria

1. Receiving mechanical ventilation or continuous positive airway pressure (CPAP).
2. Skin abnormalities in the nasopharynx and/or oropharynx.
3. Contraindication to application of skin sensors.
4. Known arrhythmia.
5. Presence of a congenital abnormality requiring major surgical intervention.
6. Any medical or psychosocial condition or circumstance that, in the opinion of the investigators, would interfere with the conduct of the study or for which study participation might jeopardize the neonate's health.

For the feasibility, usability and acceptability assessment, ETNA study HCPs and hospital administrators will be enrolled if they are 18 years or older, involved in or aware of the ETNA study, and have provided written informed consent. Caregivers may be enrolled if they are 18 years or older, have a neonate enrolled in the study, and are willing to participate in a 30-minute IDI as well as direct observation while their neonate is on or attached to the investigational devices.

3.3.3 Sample Size

A total of up to 500 neonates will be enrolled. For the verification of the reference devices, up to 20 neonates will be enrolled in the AKU-N nHDU or post-natal ward. Once this initial testing and data collection of the reference devices is complete, up to 120 neonates per investigational device will be enrolled in the AKU-N nHDU or post-natal ward for a total of 240 neonates. Enrollment will then move to PMH for the clinical feasibility phase, and up to 120 neonates per investigational device will be enrolled for a total of 240 neonates.

For the feasibility, usability, and acceptability assessment, all ETNA study HCPs and relevant hospital administrators will be asked to participate in the data collection procedures. Caregivers who consent to participate in the acceptability assessment may participate in an IDI as well as direct observation while their neonate is being monitored by the reference and/or investigational devices. Up to 30 caregivers at each site will be enrolled.

3.4 Study Period

Following enrollment, each neonate will be observed for a minimum of 1 hour and potentially for the entire duration of their stay in the hospital. A neonate may be enrolled to the study more than once as long as they meet the eligibility criteria and the caregiver(s) is willing to have their neonate participate. Projected duration of enrollment is anticipated to be about 12 months for this study.

4 STUDY PROCEDURES

Note that at study initiation, a pilot study will be conducted in up to 10 study participants. For these study participants, all study procedures as outlined below will be followed. The purpose of this pilot study is to evaluate and optimize study procedures and ensure study feasibility in a

For peer review only - http://bmjopen.bmj.com/site/about/guidelines.xhtml

situation that mirrors the clinical trial. All data collected from study participants enrolled in the pilot study will be maintained in a separate pilot study database and will not be analyzed with the study data from the main trial. The target enrollment of 500 participants for the main trial does not include the study participants enrolled in the study pilot.

Refer to Appendix I for **Study Flow Diagram** and Appendix II for **Study Procedures and Visits Table.**

4.1 Recruitment

Recruitment for this study will be performed by ETNA study staff. Neonates ≤28 days corrected age admitted to the AKU-N nHDU or post-natal ward and the PMH newborn unit will be assessed by ETNA study staff for potential screening for the study. A brief introduction to the study will be provided to the caregiver(s) to see if the caregiver is interested in learning more about the study and in potentially having their neonate assessed for eligibility.

All hospital staff involved in ETNA study recruitment procedures will be trained in relevant study-specific procedures and certified in good clinical practice (GCP). Each recruitment and referral interaction will be documented for study records.

4.2 Screening

Screening procedures are conducted by ETNA study staff to determine eligibility for enrollment in the study. All inclusion/exclusion criteria must be assessed at time of admission. The following procedures will be performed by ETNA study staff for screening:

- Provide information on the study and answer any questions.
- Assess all eligibility criteria, including if the baby is on mechanical ventilation or CPAP, skin abnormalities of the nasopharynx and/or oropharynx, any contraindications for skin sensors, arrhythmia, and presence of congenital abnormality.
- Collect de-identified demographic information.

For those neonates who are not eligible, ETNA study staff will inform the caregiver(s) that their neonate will not be able to participate in the study and will continue to receive hospital standard care. There will be no preferential treatment of study participants. All screening procedures will be documented in the appropriate study forms, including logs and case report forms. Clinical assessments and findings will also be documented in the neonate's medical record, as appropriate. No identifying information will be retained for any neonate who does not enroll in the study.

4.3 Informed Consent Process

For the purposes of this protocol, "caregiver" refers to the neonate's parent(s). Informed consent will be obtained from each neonate's caregiver to ensure that the caregiver is informed of and fully understands what will and may happen to their neonate while participating in this research study. ETNA study staff will administer a comprehension checklist to potential participants' caregivers prior to obtaining written informed consent to ensure that caregivers fully comprehend the nature of the study. The informed consent process continues throughout the study. Key study concepts will be reviewed periodically with the caregivers. Additionally, if any new information is learned that may affect the caregiver's decision to stay in the study, this information will be shared with the caregivers in writing. All consent materials will be approved

For peer review only - http://bmjopen.bmj.com/site/about/guidelines.xhtml

by the appropriate Institutional Review Boards (IRBs) prior to use. Approval will also be sought from the Kenyan Pharmacy and Poisons Board and the Kenyan National Council for Science and Technology Bioethics Committee as required by Kenyan legislation. These will be presented to county and hospital leadership for administrative approval as well.

Written informed consent will be collected from all HCPs and hospital administrators participating in the qualitative portion of the study. HCPs and hospital administrators may decline to participate without any negative effects on their employment. Caregivers may decline to participate in the qualitative portion of the study and still have their neonate participate in the primary portion of the study.

Refer to detailed description of informed consent procedures and ethics committee approval in Section 11 (Ethical Considerations and Consent).

4.4 Enrollment

After screening is complete, ETNA study staff will perform the enrollment visit procedures for those neonates who are eligible and whose caregiver(s) is willing to participate. The following procedures will be performed at enrollment:

- Obtain written informed consent for enrollment.
- Assign participant identification (ID) study number.
- Collect gestational age/corrected age, medical history, and any additional socio-demographic information not already collected during screening.
- Collect information regarding duration of pregnancy, mode of delivery, and Apgar score results.
- Collect information regarding medications.
- Place reference and/or investigational device(s) on neonate.
- Obtain baseline vital signs.

All ETNA study enrollment procedures will be documented in the appropriate study forms. Clinical assessments and findings will also be documented in the neonate's medical record, as appropriate.

For HCPs and hospital administrators who consent to participate in the qualitative portion of the study, they will undergo an IDI after they have completed at least one month of work on the study. For caregivers who consent to participate in the qualitative portion of the study, they may undergo an IDI at any time during their neonate's participation in the study, depending on when is most convenient for the caregiver. The IDI will not exceed 30 minutes and will be conducted by a trained ETNA study staff member using a questionnaire.

4.5 Observations

Observations while the investigational device(s) and reference device(s) are in use are a minimum of 1 hour. Observations will occur in the hospital while the neonate is receiving clinical care. During observations, the following procedures will be done:

- Update medical history, including clinical procedures, interventions, therapies, additional bloodwork or laboratory tests.

For peer review only - http://bmjopen.bmj.com/site/about/guidelines.xhtml

- Update information regarding medications.
- Collect information about environmental features and exposures during hospitalization.
- Check placement of reference and investigational device(s). Assess and collect information regarding reference and/or investigational device(s) removal, repositioning, or dislodgement.
- Set-up videotape recorder and/or take photographs of neonate and the device(s). The neonate's face will not be recorded or photographed. Please refer to the **ETNA Study Manual** for instructions on appropriate photography and videotape recorder placement.
- Track and record any of the following activities during monitoring:
  - Kangaroo mother care.
  - Feeding.
  - Diaper change / clothes change.
  - Repositioning of the neonate.
  - Crying.
  - Bathing.
  - Clinical procedures including bloodwork, line insertion, nasogastric tube placement, umbilical wound care, drug administration, UV therapy and/or stimulation.
- Assess for safety issues and report safety events.

4.6 Interrupted or Missed Observations

If at any time during enrollment a neonate's observation is interrupted or discontinued (i.e., due to clinical procedures, kangaroo mother care, feeding, diaper/clothes change, repositioning of the neonate, or bathing, etc.), ETNA study staff will attempt to place the reference device(s) and/or investigational device(s) back on the neonate to resume observations. Prior to attempting to place any device back on a neonate, ETNA study staff will ensure that this activity does not interfere with any clinical care or treatment of the neonate. Study staff will attempt to place the reference device (s) and/or investigational device(s) until successful for up to 30 minutes, with 30 minutes period of rest between each attempt. Any attempt at placement will be discontinued if requested by the caregiver or clinical staff.

4.7 Withdrawal and Early Termination

Neonates and their caregivers may voluntarily withdraw from the ETNA study for any reason at any time. The ETNA site investigators may also withdraw neonates from the study in order to protect their safety if, in the investigators' opinion, continuing participation would jeopardize the neonate's health. HCPs, hospital administrators, and caregivers may voluntarily withdraw from the qualitative portion of the study for any reason at any time. Any participant withdrawal or early termination will be documented in the appropriate study forms.

4.8 Study Termination

Study participation will conclude prior to discharging of the neonate from the unit. The ETNA study staff will liaise with the hospital staff to be notified of when a participant will be discharged. At the conclusion of participation, the following procedures will be conducted:

For peer review only - http://bmjopen.bmj.com/site/about/guidelines.xhtml

- Update medical history, including clinical procedures, interventions, therapies, additional bloodwork or laboratory tests.
- Update information regarding medications.
- Remove reference and investigational device(s).
- Document contact in neonate's study records.

5 STUDY DEVICES

5.1 Descriptions

This study will utilize the following reference and investigational devices:

Reference devices:

- Masimo Rad-97™ is the reference or comparator device for the following physiological measurement parameters of interest: HR, RR, SpO2, and capnography.
- Tempo Easy Bleu thermometer is the reference or comparator device that will be used to measure skin temperature.

Investigational devices:

- EarlySense system is an investigational device that will be tested in comparison to the Masimo Rad-97™ reference devices to measure the following physiological measurement parameters of interest: HR, RR, and apnea.
- ANNE system is an investigational device that will be tested in comparison to the Masimo Rad-97™ and the Tempo Easy Bleu thermometer reference devices to measure the following physiological measurement parameters of interest: HR, RR, SpO2, and skin temperature.

Further details describing each of the investigational devices and their components are detailed in section 1.3 "Investigational Devices" as well as the specific reference and investigational device standard operating procedures (SOP) documents.

5.2 Method for Assigning Participants

The reference and investigational devices will be assigned to enrolled neonates at time of enrollment based on availability of devices and, if possible, both investigational devices may be placed on the neonate simultaneously.

5.3 Placement of the Devices

Detailed instructions on how to appropriately place the Masimo Rad-97™, Tempo Easy thermometer, EarlySense system, or ANNE system components, as well as troubleshooting issues with placement or device readings, are outlined in the reference and investigational device specific SOPs: the **Masimo Rad-97™ device SOP**, **Tempo Easy thermometer SOP**, **the EarlySense system SOP**, and **ANNE system SOP**.

For peer review only - http://bmjopen.bmj.com/site/about/guidelines.xhtml

5.4 Blinding of Study

During the clinical feasibility phase, hospital and site study staff will be blinded to data collected by the investigational devices. While they will have access to the monitor or display screens for the Masimo Rad-97™ and Tempo Easy Bleu thermometer for clinical care of the enrolled neonate while in use for the study, any and all EarlySense system or ANNE system monitors or display screens, will be blank or covered fully. Data from the investigational devices will continue to be collected and recorded for analysis. The alarms and audio alerts from all devices will be disabled, however, events that may trigger an alarm will continue to be monitored and recorded.

5.5 Packaging

Details regarding packaging of the reference and investigational devices can be found in the **Masimo Rad-97™ device SOP**, **Tempo Easy thermometer SOP**, **the EarlySense system SOP**, and **ANNE system SOP**.

5.6 Receiving, Storage, Dispensing and Return

Details regarding receipt, storage, dispensing, and return of the reference and investigational devices can be found in the **Masimo Rad-97™ device SOP**, **Tempo Easy thermometer SOP**, **the EarlySense system SOP**, and **ANNE system SOP**.

6 DATA COLLECTION

All ETNA clinical research data, including data collected from the reference and investigational devices, will be maintained through a combination of secure electronic data management system and physical files with restricted access. Data related to study endpoints will be uploaded from the devices to electronic databases and then extracted from the electronic databases for statistical analysis. A separate secure database will be used to store potentially identifiable information. This database will link identifiable information to participant ID. All documentation (paper-based or electronic) that has both personal identifiers and the participant ID will have restricted access and will be stored in a secure manner separately from other study data. All ETNA databases will be retained for at least five years after the last participating neonate exits the study.

6.1 Case Report Forms

ETNA study data will be collected by ETNA study staff using designated source documents or case report forms (CRFs). ETNA study data will be entered directly into the CRFs during study observations. The neonate will only be identified in the CRF by a unique participant ID. Data from the CRFs will be entered into the electronic database as promptly as is feasible. ETNA study staff will maintain source documents for each neonate at the study site. Source documentation will be available for review to ensure that the collected data are consistent with the CRFs. CRFs, source documents and other supporting documents (both electronic and paper-based) will be kept in a secure location and remain separate from participant identification information (name, address, etc.) to ensure confidentiality. GCP will be followed to ensure accurate, reliable and consistent data collection.

For peer review only - http://bmjopen.bmj.com/site/about/guidelines.xhtml

6.2 Questionnaires

Qualitative data will be collected through IDIs and recorded on a paper questionnaire by the ETNA study staff. Similar to the CRFs, data from the questionnaires will be entered into an electronic database as promptly as is feasible. The paper copy of the questionnaire will be maintained as source document for each HCP, hospital administrator, or caregiver enrolled to the qualitative portion of the study. No identifying information will be collected on the questionnaire. Paper questionnaires will be kept in a secure location and remain separate from participant identification information (name, address, etc.) to ensure confidentiality. GCP will be followed to ensure accurate, reliable and consistent data collection

6.3 Source Documents

Source documents include but are not limited to:

- Signed informed consent forms (ICFs).
- Documentation of the comprehension checklist.
- Documentation that includes dates and times of observations.
- Clinical notes.
- Paper questionnaire.

ETNA site investigators will maintain, and store in a secure manner, all source documents throughout the study. These documents will be retained for at least five years after the last neonate exits the study.

6.4 Device Data Collection

The placement of the device sensors will be documented with photo(s) and/or video(s) for each device in each neonate. Continuous physiological data and event data will be recorded from the reference and investigational devices at least once a second. All electronic data will be collected wirelessly or via a wired connection, from the reference and investigational devices to a study laptop using custom software applications from the device developers. The laptop will be backed-up to a secure sever at AKU-N on a daily basis. Data will be identified with unique file names containing the date and participant ID. No personal identifying information will be collected by either the reference or investigational devices.

6.5 Data Management

Local ETNA data management will take place at AKU-N, with support from SCUS. Data management activities include transferring reference and investigational device data, CRF data entry and validation, data cleaning, database quality control (QC), disaster recovery plans, preparation and submission of compliance reports to the funding agency, and preparation of the final study database. De-identified reference and investigational device data will be uploaded from the secure server to a secure electronic data management system to support data cleaning and statistical analyses. All transfer of data for analysis will use end-to-end encryption with two–factor authentication. An audit trail will be maintained for any de-identified data leaving AKU-N for analysis.

6.6 Data Access

The ETNA participating study sites will maintain appropriate medical and research records for this study, in compliance with GCP, regulatory, sponsoring organization and institutional

For peer review only - http://bmjopen.bmj.com/site/about/guidelines.xhtml

requirements for the protection of confidentiality of neonates. De-identified data will be provided to the investigators to facilitate data cleaning and analysis. De-identified reference data and device developer specific data will be provided to each device developer to facilitate device improvement and to ensure that the device developer data is synchronized with the reference data. The site will permit authorized representatives of the sponsor and regulatory agencies to examine (and when required by applicable law, to copy) clinical records for the purposes of quality assurance reviews, audits and evaluation of the study safety and progress. User rights will be provided to ETNA study staff, PIs, and co-investigators and the investigational device developers at the level appropriate for each individual's job description.

6.7 Data Storage

The ETNA site investigators and designees will maintain, and store securely, complete, accurate and current study records throughout the study. ETNA study staff will retain all study records on site for at least five years after study closure. Study records will not be destroyed prior to receiving approval for record destruction from the sponsor. Applicable records include source documents, ICFs, and notations of all contacts with the participating neonate and caregivers.

At the completion of the study, de-identified data will be transferred to a public data repository to share with other internal and external researchers.

7 SAFETY ASSESSMENT AND MONITORING

7.1 Safety Monitoring

The study site investigators will be responsible for close safety monitoring of all neonates participating in the study, and for alerting the ETNA protocol team if unexpected concerns arise. All neonates will be carefully screened to ensure that they do not demonstrate any exclusion criteria.

7.2 Adverse Device Effect

For the purposes of this study, an ADE is defined as the following: any untoward and unintended response to a medical device; this includes any event resulting from insufficiencies or inadequacies in the instructions for use or the deployment of the device; this includes any event resulting from a user error; this includes patients and users. Where the definition indicates "device", it refers to any device used in the study. This might be the device under investigation, or any market released component or instrument of the system used to implant the device.

The ETNA protocol team anticipates ADEs and SAEs to occur among enrolled neonates at a similar rate as untoward medical events occur in comparable pediatric populations outside of a research setting. ADEs that the study team expects may occur during the research include, but are not limited to: adverse reactions to the reference and investigational device sensors (e.g., erythema or edema at the sensor site). The site investigators will be assessing all ADEs resulting in skin damage and treat or refer the participating neonate for medical care as appropriate, which may include removal of the device(s) and withdrawal of the neonate from the study if necessary. If any acute treatment or medical care is required as a result of harm caused by investigational and/or reference devices or study procedures, this care will be

For peer review only - http://bmjopen.bmj.com/site/about/guidelines.xhtml

provided by the site free of charge. All neonates in the study with an ADE resulting in skin damage will be followed clinically until the ADE resolves (returns to baseline) or stabilizes.

7.3 Serious Adverse Events

A SAE will be defined as any ADE resulting in permanent skin damage..

7.4 Adverse Event Relationship to Devices

The relationship of ADEs (skin damage) to the investigational or reference device(s) will be assessed as follows:

- Definitely related: ADE and administration of the device are related in time, and a direct association can be demonstrated with the device.
- Probably related: ADE and administration of the device are reasonably related in time, and the ADE is more likely explained by the device than by other causes.
- Possibly related: ADE and administration of the device are reasonably related in time, and the ADE can be explained equally well by causes other than the device.
- Probably not related: a potential relationship between administration of the device and ADE could exist, but is unlikely, and the ADE is most likely explained by causes other than the device.
- Not related: the ADE is clearly explained by another cause unrelated to administration of the device. Reportable events must have documentation to support the determination of “not related.”

The initial determination of ADE relationship to the device will be made by study staff with as needed consultation with the local site investigators. An independent medical monitor will review determinations of ADE relationship and assign the final relationship determination, including all SAEs.

7.5 Grading Severity of Events

Any ADE resulting in erythema or edema that does not resolve within 12 hours will be assessed and reported.

Skin condition at the sensor site will be assessed based on severity of erythema and edema, as well as skin breakdown.

7.6 Safety Reporting

All ADEs and SAEs must be reported by the site to the study medical officer and sponsor within 24 hours. Photographs of ADE will be taken and included in the report. Attribution with regard to relationship to the device will only be reported for all ADEs and SAEs. Any ADE described by the site staff as probably, or definitely related to the device requires immediate notification by the site study staff to the medical officer, co-PIs, and sponsor. These ADE cases will be forwarded to an independent medical monitor for review and the independent medical monitor will make the final determination of relationship to the study device. The independent medical monitor, study medical officer, co-PIs, and sponsor will convene within 24 hours by teleconference and decide whether the event necessitates a pause in further enrollment.

. Reporting requirements for the IRB will be followed as appropriate.

For peer review only - http://bmjopen.bmj.com/site/about/guidelines.xhtml

8 TRAINING REQUIREMENTS, MONITORING AND REPORTING

8.1 Training Requirements

All ETNA study staff will be trained in the Protection of Human Subjects and GCP prior to any interactions with study participants. Prior to study initiation, all ETNA study staff will receive training on all study procedures, including the study protocol, SOPs, data collection tools, informed consent process and reporting. Trainings will be conducted by a representative of the study sponsor or study consortium or other qualified clinician, as appropriate for the training material. ETNA study staff involved in placement and handling of the devices will be trained by qualified individuals from the sponsor or device developers.

8.2 Monitoring

The ETNA study site investigators will be responsible for close safety monitoring of all neonates participating in the study, and for alerting the ETNA protocol team if unexpected concerns arise. All neonates will be screened carefully prior to enrollment to ensure that neonates do not demonstrate any exclusion criteria. This study does not involve any direct diagnosis, treatment, or intervention, so each participating neonate will be cared for per local standard of care by hospital staff, independent of ETNA study activities.

ETNA study investigators will hold regular conference calls to monitor progress and ensure homogeneity and safety in protocol execution.

8.3 Study Discontinuation

The ETNA study may be discontinued at any time by the ETNA protocol team, funding agency, regulatory authorities, or IRBs.

9 STATISTICAL DESIGN AND ANALYSIS

9.1 Overview and General Design

The goal of the ETNA study is to determine the accuracy and clinical feasibility of two investigational non-invasive continuous physiological monitoring devices in neonates in a LRS. We will initially perform verification of the reference devices in a cohort of 20 neonates. We will determine repeatability using within subject repeated observations of the relevant parameters of interest. The reference device measurements will be compared to manual measurements, clinical monitor observations, and video-assisted observations.

We will then asses accuracy of the investigational devices by measuring the agreement between the reference device(s) and each investigational device using repeated observations in a new cohort of 20 neonates. Agreement between the reference and investigational device(s) will be determined using an *a priori*-defined non-inferiority threshold. Thresholds will be determined based on the repeated observations performed on the reference device in the verification phase, international standards and clinical expert consensus opinion. Two *a priori*-determined thresholds will be determined: one lower threshold that would allow the device developer to optimize the device for retesting, and a second higher threshold that will allow the device to move on to the clinical feasibility phase of testing. A maximum of five rounds of testing and retesting will be permitted for each investigational device. Each round of testing or

For peer review only - http://bmjopen.bmj.com/site/about/guidelines.xhtml

retesting will consist of using an additional cohort of 20 neonates. Should the lower threshold not be reached for at least one parameter, no further testing of the investigational device will be performed.

An investigational device will advance to the clinical feasibility phase once the agreement for the measurement parameters of interest exceed the higher non-inferiority threshold. The clinical feasibility phase will be performed in a less-controlled environment at PMH in a cohort of up to 120 neonates for each device. We will evaluate the agreement between repeated observations from the reference device(s) and the investigational device(s) as well as diagnostic performance in clinical event detection. Additional performance metrics such as alarm rates, alarm delays and uptime\downtime will be compared between the reference and investigational devices.

9.2 Analytical Methodology

Every second of data will be automatically graded according to the quality of the data for each measurement parameter of interest. The quality will be graded as *optimal, acceptable* and *unacceptable* based on predefined rules for each device and each measurement parameter of interest. The Masimo Rad-97™ provides a signal quality index that will be used to determine data quality. Each of the investigational devices also provide a signal quality index. The quality thresholds will be determined following the verification phase. All comparisons will be performed from observations between two devices (or a single device during the verification phase). We will randomly select multiple (a least 10) observations of 120 seconds of optimal quality data in each neonate, at least five minutes apart, for each measurement parameter of interest from the full recording. For the clinical feasibility phase, accuracy comparisons will use optimal or acceptable data. We will use at least three hours of recording to a maximum of 12 hours for the performance metrics such as alarm rates, alarm delays and uptime\downtime.

The repeatability of the reference device parameter estimates initially will be assessed with the intraclass correlation coefficient (ICC). Additional training or standardization of procedures will be performed to ensure at least good repeatability (ICC >0.7). This will be followed by measuring agreement between the repeated reference observations and between the manual, clinical monitor and video-assisted methods and the reference observations using the methods described by Bland and Altman for replicated observations.[6] The agreement will be reported as a mean bias and limits of agreement with 95% confidence intervals (CIs). Graphical representation of the data will be assessed with agreement plots, Clark error grids, and Polar plots to identify extreme outliers and significant data trends. We will extend the reliability analysis using inter-rater and intra-rater reliability with an equivalence test for agreement.[7]

In the accuracy evaluation, we will calculate the root mean square difference (RMSD) and ICC for each measurement parameter of interest to compare the multiple repeated observations between the reference and investigational devices. We will then calculate the agreement between the reference device(s) and each investigational device using the methods described by Bland and Altman for replicated observations. The agreement will be reported as a mean bias and limits of agreement with 95% CIs. Graphical representation of the data will be assessed with agreement plots, Clark error grids, and Polar plots to identify extreme outliers, impact on clinical decisions, and significant data trends. We will use an *a priori*–defined non-inferiority margin for agreement as a threshold value to allow for decisions regarding proceeding to additional testing.

For peer review only - http://bmjopen.bmj.com/site/about/guidelines.xhtml

In the clinical feasibility phase, agreement between the reference device(s) and each investigational device will be assessed as in the accuracy evaluation phase. Event detection rates, alarm rates, alarm delays and uptime/ downtime will be summarized with means, medians, standard deviations and intra-quartile ranges as appropriate. Summaries of sensitivity, specificity, positive predictive values and negative predictive values comparing each measurement parameter of interest in the investigational device(s) to the reference device(s) will be produced. Comparisons of binary events will be assessed using Cohen's weighted Kappa and McNemar's test. The non-inferiority of alarm rates, alarm delays and uptime/ downtime will be evaluated based on predefined inferiority margins.

Qualitative data will be collected through IDIs and/or semi-structured questionnaires and analyzed to assess feasibility, usability and acceptability of the investigational devices among HCPs and hospital administrators, and acceptability among caregivers of enrolled neonates. The qualitative data will be in narrative format and the results will be descriptive. The questionnaires will be coded and analyzed using a codebook with identified themes, including feasibility of using each investigational device, barriers and facilitators to use, and perceived value. Qualitative data analysis software will be used to organize, code, and analyze the qualitative data in an iterative process. The research team will start by identifying an initial set of codes and themes based on the categories from the IDI guides. During the coding process, attention will be paid to identifying emergent issues and themes that will be added to the codebook and included in the analysis. Responses from the IDIs will be coded and discrepancies will be discussed and resolved for the final analysis and theme identification.

9.3 Sample Size Estimation

Sample size estimates for method comparison studies typically depend on the confidence interval required around the limits of agreement. Sample sizes of 100-200 typically provide a tight confidence interval. The standard deviation of the difference between the reference and investigational devices for RR (predicted to be the widest standard deviation of the parameters of interest measured) is estimated to be three breaths per minute for optimal data, and five breaths per minute for (to be adequate data obtained in the clinical feasibility phase at PMH).

Verification sample size: 20 neonates with 10 replications per neonate will give the 95% CI of limits of agreement between the reference device(s) and the standard clinical measurements to be +/- 0.76 times the standard deviation of their differences. This would be approximately 2.3 breaths per minute based on a standard deviation of three breaths per minute.

Accuracy sample size: 20 neonates with 10 replications per neonate per device will give the 95% CI of limits of agreement between the reference device(s) and the investigational device to be +/- 0.76 times the standard deviation of their differences.

Clinical feasibility sample size: 100 neonates per device will give the 95% CI of limits of agreement between the reference device(s) and the investigational device to be +/- 0.34 times the standard deviation of their differences. For event detection comparison (based on a hazard ratio of 0.75), 379 events will provide 80% power to detect a 25% difference in incidence rate at 5% significance level, assuming that the same numbers of neonates are tested by the reference device(s) and the investigational device. This is assuming we collect one event per hour for 3-4 hours of recordings in each of the 100 neonates.

For peer review only - http://bmjopen.bmj.com/site/about/guidelines.xhtml

10 RESULT PRESENTATION

Results of this research may be presented through published manuscript(s) with detailed description of the background, methods, results, discussion and conclusions. The specific format and details of any potential manuscript will be in accordance with the requirements of the publishing journal.

10.1 Dissemination of Results

The results of this study may be published collaboratively by ETNA investigators at SCUS, in peer-reviewed journals. ETNA study findings will be presented to staff at each study site. ETNA study results may be presented at international conference(s) to disseminate the findings of the study.

11 ETHICAL CONSIDERATIONS AND CONSENT

11.1 Principles for Clinical Research

This clinical study will be conducted in compliance with the protocol, GCP, and all applicable regulatory requirements and IRB reviews. All ETNA study activities will follow the ethical principles of the Declaration of Helsinki. All ETNA study staff will be trained and certified in the protection of human subjects.

11.2 Institutional Review Boards and Independent Ethics Committees

The IRB of record for this study is AKU-N Research Ethics Committee. A copy of the protocol, proposed ICFs, other written participant information, and any proposed advertising materials will be submitted to AKU-N Research Ethics Committee for written approval. The protocol will be submitted to Western Institutional Review Board (WIRB) as well for review and approval. The ETNA investigators must submit and, where necessary, obtain approval from the IRB at their local institution for the initiation of the study and all subsequent protocol amendments and changes to the ICF. SCUS is responsible for assuring that this protocol, ICFs and any other study-related documents are approved by AKU-N Research Ethics Committee prior to implementation of the protocol. Any subsequent amendments to the protocol or other study-related documents must be approved by AKU-N Research Ethics Committee prior to implementation. The ETNA study will be conducted in full compliance with the protocol. Any deviations from or violations of the protocol will be documented and submitted to the appropriate IRB by investigators as required. The protocol will not be amended without prior written approval by the SCUS ETNA PI.

11.3 Informed Consent Documentation

In obtaining and documenting informed consent, the ETNA site investigators and their designees will comply with applicable local and domestic regulatory requirements and will adhere to GCP. English and Swahili versions of the ICF will be reviewed and approved by the appropriate IRBs before use with participant neonates' caregivers. The ICF will include the purpose of the study, a description of the procedures to be followed and the risks and benefits of participation. The informed consent process will give caregivers all of the relevant

For peer review only - http://bmjopen.bmj.com/site/about/guidelines.xhtml

information necessary to decide whether to participate, or to continue participation, in this study. Potential research participant neonates' caregivers will be permitted to ask questions and to exchange information freely with the ETNA study team. If the caregiver providing consent is illiterate, an independent witness will be present to verify to the caregiver that all the information read aloud is contained in the ICF. In this instance, the caregivers will thumbprint the ICF, which will be countersigned by the impartial witness.

Before a neonate begins participation in the study, it is the ETNA site investigators' responsibility to ensure that informed consent is obtained from their caregiver after adequate explanation of the aims, methods, and potential risks and benefits of the study. The ETNA study staff obtaining consent will also sign and date the ICF. A signed and dated copy of the consent form will be given to the participant neonate's caregiver and this will be documented in the participant neonate's study record.

Before a HCP, health administrator, or caregiver begins participation in the qualitative portion of the study, the ETNA site investigators will ensure informed consent is obtained after adequate explanation of the aims, methods, and potential risks and benefits of the study have been provided to the participant. The ETNA study staff obtaining consent will also sign and date the ICF. A signed and dated copy of the consent form will be given to the participant and this will be documented in the participant's study record

11.4 Risks and Benefits

11.4.1 Risks to Participants

Coercion
Caregivers may feel coerced tor compelled to enroll in the study in order for their neonate to receive care within a research setting, which may be perceived as a higher quality than the standard of care.

Medical Management
Participation in the study has the potential to compromise a neonate's inpatient care if study procedures are prioritized. ETNA study staff will guarantee that this will not be the case, and neonates may be excluded if study staff believes that including them in the study could jeopardize their medical care in any way.

Reference and Investigational Devices
Placement and attachment of the reference and investigational devices may cause the neonate minor, temporary distress. Placement of the investigational devices (e.g. physical placement or timing of placement) has the potential to delay clinical care. ETNA study staff will guarantee that this will not be the case, and neonates may be excluded if study staff believes that including them in the study could jeopardize their medical care in any way.

11.4.2 Protection against Risks

Coercion
During the informed consent process, ETNA study staff will emphasize that the study is optional and strictly voluntary, and that the neonate will receive medical care whether enrolled in the study or not.

For peer review only - http://bmjopen.bmj.com/site/about/guidelines.xhtml

Medical Management

In order to minimize the possibility that participation in this study will interfere with the standard medical management of neonates, ETNA study staff will be trained in integrating research procedures with clinical care. **Clinical care will always be prioritized above research procedures.**

Reference and Investigational Devices

ETNA study staff will be trained in the appropriate placement of the reference and investigational devices' sensors to minimize discomfort to the neonates as well as to avoid interference with any assessment, treatment, or intervention necessary for clinical care.

11.4.3 Benefits to Participants

There is no direct benefit to neonates enrolled in this study.

11.5 Participant Confidentiality

The ETNA site investigators must ensure that the neonate's confidentiality is maintained. Personal identifiers will not be included in any study reports. All study records will be kept confidential in keeping with IRB regulations as well as national and local laws. Video recordings and photographs will not include the neonate's face or caregiver's face. All study procedures will be conducted in such a manner as to protect participant privacy and confidentiality to the fullest extent possible.

12 POSSIBLE CONSTRAINTS

Anticipated implementation challenges to the successful outcome of the study include:

- Delays in device development and evaluation if the investigational device developers encounter engineering or equipment issues. Regular communication between the co-PIs, co-investigators and device developers along with close monitoring of study progress will help to anticipate, prepare for and mitigate potential engineering or equipment issues that would cause delays in device development and evaluation.
- Coordinating and standardizing certain procedures across the different sites, devices, and data platforms. A study manual and SOP documents will be developed to provide clear and detailed instructions on study procedures, devices, and data management activities. Standardized training will be provided.
- Ensuring quality and consistency of implementation across the different sites. Standardized training, supervision, and oversight will be provided to ensure quality and harmonized trial procedures.
- Difficulty in recruitment of neonates. This study will be conducted at high-volume neonatal wards and maternity wards to maximize enrollment. Sensitization and outreach activities will also be conducted to aid with recruitment.
- Perception of unequal care by non-ETNA caregivers. Study participant neonates will receive the same standard clinical care as all other neonates in the nHDU, post-natal ward, and neonatal unit. Sensitization sessions with both ETNA study caregivers and non-ETNA study caregivers will be conducted to provide information about the study and answer questions.

For peer review only - http://bmjopen.bmj.com/site/about/guidelines.xhtml

- Difficulties with application of reference and investigational devices. Study staff will be carefully trained on how to place the reference and investigational devices appropriately and in a manner that will minimize any possibly agitation of the neonate or cause discomfort for the neonate. Reference device(s) and/or investigational device(s) will be removed if requested by a caregiver or by hospital staff.
- Appropriate investigational device set-up and maintenance. Wires for the investigational devices should not be loose, to prevent tripping and falling. Study staff will be trained on proper installation procedures for the investigational devices.
- Limited research resources at AKU-N and PMH. AKU-N is receiving funding from the United States National Institutes of Health for research administration capacity building as well as research training and mentorship from the University of Washington, Seattle, WA, USA. PMH staff involved in ETNA will participate in all relevant research and study-specific training via AKU-N. Study progress will be monitored at both sites on a weekly and monthly basis to identify areas that require additional support.

PROTOCOL REFERENCES

United Nations Inter-agency Group for Child Mortality Estimation (UN IGME), 'Levels & Trends in Child Mortality: Report 2018, Estimates developed by the United Nations Inter-agency Group for Child Mortality Estimation' United Nations Children's Fund, New York, 2018.

[2] Sustainable Development Goals Knowledge Platform. Division for Sustainable Development Goals.[website]. New York, NY: United Nations Headquarters. Date Accessed: November 27, 2018. https://sustainabledevelopment.un.org/sdg3

[3] Zhu Z, Liu T, Li G, Li T, Inoue Y. Wearable sensor systems for infants. *Sensors (Basel)*. 2015;15(2):3721-49. Published 2015 Feb 5. doi:10.3390/s150203721

[4] Sahni R. Continuous noninvasive monitoring in the neonatal ICU. Current Opinion in Pediatrics. 29(2):141–148, Published 2017 Apr 1. Doi: 10.1097/MOP.0000000000000459

[5] https://www.cardinalhealth.com/en/product-solutions/medical/patient-recovery/patient-care/obstetric-and-neonatal/neonatal-care/baby-hydrogel-adhesive-tape.html

[6] Bland JM, Altman DG. Measuring agreement in method comparison studies. Stat Methods Med Res. 1999;8(2):135–60.

[7] Yi, Q. , Wang, P. P. and He, Y. (2008), Reliability analysis for continuous measurements: Equivalence test for agreement. Statist. Med., 27: 2816-2825. doi:10.1002/sim.3110

For peer review only - http://bmjopen.bmj.com/site/about/guidelines.xhtml

APPENDICES

Appendix I: Schedule of Study Procedures and Evaluations

Activity	Screening	Enrollment	Observation	Discharge
Eligibility assessment	X			
Informed consent and comprehension checklist	X			
Assign participant ID	X			
Demographics	X	X		
Medical history		X	X	X
Maternal pregnancy history		X		
Medications use		X		
Placement of reference and/or investigational device(s)		X	X	
Collection of vital signs		X	X	X
Video tape recording and/or photographs			X	
Track clinical care and non-study activities			X	X
Safety assessment			X	
End of study questions				X
Removal of reference and/or investigational device(s)				X

For peer review only

For peer review only - http://bmjopen.bmj.com/site/about/guidelines.xhtml

Appendix II: Informed Consents

Informed Consent Form for Enrollment [Newborn caregiver]

Evaluation of Technologies for Neonates in Africa (ETNA) Study
Version 1.1, 18 June 2019

Protocol title: Evaluation of Technologies for Neonates in Africa (ETNA) project

Sponsor: Save the Children Federation, Inc., United States of America

LOCAL PRINCIPAL INVESTIGATORS:
Dr. William Macharia
Aga Khan University, 3rd Parklands Avenue,
P O Box 30270, Nairobi
Contact phone: 254-20-3661017

For peer review only

Introduction

You are being asked for your baby to take part in this study because your baby is less than 28 days and has been admitted to this hospital. This study is sponsored by Save the Children, an organization that promotes children's rights, provides relief and helps support children worldwide. The person in charge of this study at this hospital is Dr. William Macharia.

This is a consent form that gives you information about the study and what you or your baby will have to do if you agree to be in the study. You are free to ask questions about the study at any time. If you agree to take part in this study, you will be asked to sign this consent form or make your mark/thumbprint in front of a witness. You will be given a copy of this form to keep. Another copy will stay with the study records.

Your participation is completely voluntary. You have the right to refuse to join or withdraw from the study at any time without negative consequences to you or your baby. Before you decide, you can talk to anyone you feel comfortable with about the research. If there is anything that you do not understand about the study, please ask the study staff or Dr. Macharia at any time.

Why is this Study Being Done?

In Kenya, there is a need for safe and efficient newborn care technologies that are non-invasive, low-cost, and can operate without constant operation by a healthcare provider. We would like to adapt and test devices that can repeatedly monitor a patient’s vital signs (such as heart rate, breathing, and temperature) and health status so that they can be used to reliably monitor a newborn baby in the hospital. The goal of this study is to develop and test these monitoring devices, ultimately leading to improved clinical outcomes for newborn babies.

The Bill & Melinda Gates Foundation is providing funds for this study to take place. A total of up to 500 babies admitted to Aga Khan University Hospital and Pumwani Maternity Hospital will

For peer review only - http://bmjopen.bmj.com/site/about/guidelines.xhtml

join this study. Each baby may be in the study for a minimum of 1 hour and up to the entire time they are in the hospital.

What Do We Expect to Learn From This Study?

From this study we expect to learn how to better develop safe, efficient, and non-invasive newborn care technologies for places like Aga Khan University Hospital and Pumwani Maternity Hospital.

What Do I Have To Do If I Take Part in the Study?

If you agree for your baby to be in the study, your baby may be observed for the entire time they are in the hospital starting from the time your baby is enrolled. The study staff will not be in charge of your baby's medical care and will not give you or your baby any study treatments or medicine. The study activities involve observing your baby and asking you questions about you and your baby. No study activities will be done on your baby before they have been fully explained to you, you have let us know that you understand the enrollment process and you have signed or made a mark/thumbprint on this form. Only after you read (or have read to you), discuss, and sign or make a mark/thumbprint on this form will your baby be enrolled in this study.

Study Enrollment

Upon enrollment, study devices may be placed on your baby to collect information on your baby's vital signs and movements. The study devices do **not** provide any type of care for your baby and cause no significant risk or harm to your baby. The hospital healthcare providers, and not the study staff, will be responsible for your baby's medical care, although a study nurse may help with providing routine care whenever such need arises on request of the hospital staff. Study devices may stay on your baby for the entire time they are in the hospital but will not interfere with normal care of your baby. Photographs and/or videos of your baby may be taken **but will not include your baby's face.** We will confirm with you before taking any photographs or videos of your child. During your baby's time in the study, they will continue to receive care from the hospital healthcare providers without interruption. For example, if the hospital doctor feels that your baby requires medication or body to body contact with you (Kangaroo mother care), your baby will be able to receive this recommended care even if they are in this study.

At enrollment, you will be asked to do the following things if you decide you want your baby to be in the study:

- Sign this form or make your mark/thumbprint on it after you have read it (or have it read to you), understand the study, and had the chance to ask questions about the study.
- Tell the study staff about your family's socio-demographics (like your education and income).
- Tell the study staff how long you were pregnant with your baby.
- Tell the study staff how your baby was delivered (vaginal birth or cesarean section).
- Tell the study staff if your baby is a twin or a triplet.
- Tell the study staff about any medical problems your baby has had.
- Your baby's weight will be recorded by a study team member.

For peer review only - http://bmjopen.bmj.com/site/about/guidelines.xhtml

- Your baby's vital signs will be monitored with study devices and these study devices may be placed on your baby.
- Clinical activities that take place while your baby is in the study (like medication or kangaroo mother care) will be documented.

Alternative to study participation
You have the option to not participate in this study. There will be no consequences to you or your baby if you choose not to participate in this study. Should you choose not to participate, your baby will continue to receive the normal clinical standard of care from the hospital healthcare providers.

Why Would The Doctor Take My Baby Out of This Study Early?
The study doctor may need to take your baby out of the study early if:

- The study is stopped by the sponsor, funder, ethics committee or any other regulatory body.
- If your baby is placed on mechanical ventilation or any device that assists with breathing.
- If your baby is discharged from the hospital.
- Other reasons that may prevent you and/or your baby from completing the study successfully.

What Are the Risks of Being in the Study?

- Answering questions may make you feel nervous or uncomfortable. You are free to skip any questions that you do not want to answer.
- Caregivers may feel pressured to enroll in the study in order to receive care for their baby within a research setting, which may be seen as a higher quality than the standard of care.
- Participation in the study has the potential to affect a newborn baby's inpatient care, if study procedures are prioritized. Study staff will make sure that this will not be the case, and babies may not be enrolled in the study if study staff believes that including them in the study could delay or interrupt their prompt medical attention in any way.
- Placement and attachment to the study devices may cause the newborn baby minor, temporary distress or discomfort. Placement of the study devices (e.g. physical placement or timing of placement) may also delay clinical care. Study staff will make sure that this will not be the case, and babies may not be enrolled in the study if study staff believes that including them in the study could delay or interrupt their prompt medical attention in any way.

Are There Benefits To Taking Part In This Study?
Your baby will not receive any additional benefit from being in this study. This study is designed to help understand how to develop safe, efficient, and non-invasive newborn care technologies for places like Aga Khan University Hospital and Pumwani Maternity Hospital. We hope that this technology will help babies like yours in the future.

For peer review only - http://bmjopen.bmj.com/site/about/guidelines.xhtml

What About Confidentiality?

All possible measures will be made to keep your and your baby's personal information private. We cannot guarantee 100% confidentiality. If this study is published, your name and/or your baby's will not be used and you and/or your baby will not be personally identified. Any photographs or videos of your baby will not include your baby's face.

In order to make sure the study is being done properly, your records may be reviewed by:

- Study staff and monitors
- Ethics Committee and/or Institutional Review Board (IRB)
- Save the Children
- University of British Columbia
- The device developers (EarlySense and Sonica)

Your baby's study records will be kept at the clinic/hospital for at least five years after the study is completed or for the duration required by Kenyan law. You may see your baby's records if you want to. If you decide to leave the study, information already collected from your baby will still be used for the study.

What Are The Costs To Me?

There is no cost to you for participation in this study.

Will I Receive Any Payment?

You will not receive any payment for participation in this study.

What Happens If My Baby Is Injured?

It is unlikely that your baby will be injured as a result of being in this study. If your baby is injured due to being in this study, your baby will be given immediate treatment. Contact Dr. William Macharia at 254-20-3661017 and he will tell you where your baby can get treatment.

What Are My Baby's Rights As A Research Participant?

Having your baby take part in the study is completely up to you. It is your choice. You may choose to have your baby stop the study procedures at any time – there will be no penalty or loss of benefits to which you/your baby are otherwise entitled. You and your baby will be treated the same no matter what you decide. If you choose to not have your baby be in the study, you and your baby will not lose the benefit of services to which you would normally have at this clinic and there will be no penalty or loss of benefits to which you/your baby are otherwise entitled.

We will tell you about new information from this or other studies that may affect your baby's health, welfare or willingness to stay in this study. If you want the results of the study, let the study staff know that you would like them.

A description of this clinical trial will be available on http://www.ClinicalTrials.gov, as required by United States of America law. This website will not include information that could identify

For peer review only - http://bmjopen.bmj.com/site/about/guidelines.xhtml

For peer review only

you and/or your baby. At most, the website will include a summary of the results. You can search this website at any time.

The research study was reviewed and approved by the Western Institutional Review Board in Washington, USA; the Aga Khan University Research Ethics Committee; the Pharmacy and Poisons Board; and the National Council for Science and Technology - National Bioethics Committee. This approval does not mean that the study is safe or that the committees approved your baby's participation.

What Do I Do If I have Problems or Questions?

For questions, concerns or complaints about the study, baby's rights as a research participant, or if your baby has a research-related injury, you should contact:

- Dr. William Macharia, Aga Khan University Hospital, 3rd Parklands Avenue. Tel: 254-20-3661017; email: william.macharia@aku.edu

SIGNATURE

I have read the informed consent (or had it read and explained to me), and all my questions have been answered. I have let the study staff know that I understand and I agree for my baby to take part in this study. My signature or mark/thumbprint below documents my consent.

____
Name of Participant
(Parent/Legal Guardian to print participating baby's name)

____________________ Name of Parent/Legal Guardian (print name)	____________________ Parent/Guardian's Signature and Date
____________________ Name of Study Staff Conducting Consent Discussion (print name)	____________________ Study Staff Signature and Date
____________________ Name of Witness (print name)	____________________ Witness's Signature and Date

(As appropriate, if Parent/Legal Guardian is illiterate)

For peer review only - http://bmjopen.bmj.com/site/about/guidelines.xhtml

Fomu ya maelezo na makubaliano kwa usajili (Kwa wenye kuhudumia watoto wachanga)

Utafiti wa kutathimini/kuangalia kwa undani Teknolojia za watoto wachanga barani Africa (ETA)

Toleo la 1.1, 18 June 2019

Kichwa cha Protokali: Mradi wa kutathmini/kuangalia kwa undaniTeknolojia za watoto wachanga barani Africa (ETA)

Mdhamini: Save the Children Federation Inc., United States of America

MTAFITI MKUU HAPA INCHINI:
Dr. William Macharia
Aga Khan University, 3rd Parklands Avenue,
P O Box 30270, Nairobi
Contact phone: 254-20-3661017

Utangulizi

Tunakuomba ukubali mtoto wako ashiriki kwenye utafiti huu kwa sababu ana umri wa chini ya siku 28 na amelazwa katika hospitali hii. Utafiti huu unadhaminiwa ni shirika la Save the Children, hili ni shirika ambalo linakuza haki za watoto, hutoa misaada na pia husaidia watoto duniani kote. Msimamizi wa utafiti huu katika hospitali hi ni Daktari William Macharia.
Hii ni fomu ya maelezo na makubaliano ambayo inakupatia maelezo kuhusu utafiti wenyewe na vile wewe ama mtoto wako atatakiwa kufanya ukikubali ajiunge na utafiti huu, Uko huru kuuliza maswali kuhusu utafiti huu wakati wowote. Ukikubali kushiriki kwenye utafiti huu, tutakuomba uweke sahihi yako kwenye fomu hii ya makubaliano au alama ya dole gumba mbele ya shahidi wako. Utapewa kopi ya nakala hii uweke. Kopi ingine itabaki kama rekodi za utafiti.

Kushiriki kwako kwenye utafiti huu ni hiari yako kabisa. Uko na haki ya kukataa kushiriki ama kujitoa kutoka kwenye utafiti huu wakati wowote bila ya matokeo yoyote mabaya kwako ama kwa mtoto wako. Kabla ya kuamua kushiriki ama kutoshiriki unaweza kuzungumza na mtu yeyote ambaye uko huru naye kuhusu utafiti huu. Kama kuna kitu chochote ambacho hukielewi kuhusu utafiti huu, tafadhali muulize **mfanyi kazi kwenye utafiti huu** ama Daktari Macharia wakati wowote.

Ni kwa nini Utafiti Huu Unafanywa?

Katiki inchi ya Kenya, kunahitajika teknolojia zisizoingiliana ambazo ni salama, zenye ufanisi na zenye gharama ya chini za kutunza watoto wachanga, ambazo zinaweza kufanya kazi bila usaidizi wa mara kwa mara kutoka kwa muhudumu wa afya. Tungependa kujaribu na kutumia vifaa ambavyo vinaweza kufuatilia mara kwa mara ishara muhimu za kiafya (kama vile mpigo wa moyo, kupumua, na joto mwilini) na hali ya afya ili ziweze kutumika kufuatilia kikamilifu mtoto

For peer review only - http://bmjopen.bmj.com/site/about/guidelines.xhtml

mchanga hospitalini. Lengo la utafiti huu ni kuimarisha na kujaribu vifaa hivi vya kufuatilia hali ya mgonjwa, ambavyo vitaboresha matokeo ya kiafya kwa watoto wachanga.

Taasisi ya Bill na Melinda Gates inatoa ufadhili wa kifedha ili utafiti huu ufanyike. Jumla ya watoto 500 waliolazwa katika hospitali ya Chuo Kikuu cha Aga Khan na hospitali ya kujifungulia akina mama ya Pumwani watajiunga na utafiti huu. Kila mtoto atakuwa kwenye utaiti huu kwa muda wa kati ya saa moja na muda wote ambao atakuwa bado amelazwa hospitalini.

Ni kitu gani ambacho tunatarajia kujifunza kutokana na utafiti huu?

Kutokana na utafiti huu tunatarajia kujifunza jinsi ya kubuni teknolojia zisizoingiliana za kumtunza mtoto mchanga ambazo ni salama na zenye ufanisi zitakazotumika mahali kama hapa Aga Khan University hospital na hospitali ya kujifungulia akina mama a Pumwani.

Ni kitu gani ambacho nitatakiwa kufanya nikiamua kushiriki kwenye utafiti huu?

Ukikubalia mtoto wako ashiriki kwenye utafiti huu, mtoto wako ataangaliwa kwa muda wote ambao atakuwa hapa hospitalini mara tu atakaposajiliwa kwenye utafiti huu. **Wafanyikazi kwenye utafiti huu hawatajihusisha na matibabu ya mtoto wako na hawatakupatia wewe au mtoto wako matibabu yoyote ya kiutafiti au madawa. Shughuli za utafiti zinahusisha kumuangalia mtoto wako na kukuuliza maswali kuhusu wewe mwenyewe na mtoto wako.** Hakuna shughuli zozote za utafiti zitakazofanyiwa mtoto wako bila wewe kuelezwa kikamilifu kuzihusu, utatuambia kama umeuelewa mchakato/taratibu ya kusajiliwa kwenye utafiti huu na umeweka sahihi yako au alama ya dole gumba kwenye fomu hii ya kukubali kushiriki utafiti. Mtoto wako atasajiliwa kwenye utafiti huu tu iwapo/kama utaisoma (ama kusomewa), kuijadili, na kuweka sahihi yako au alama ya dole gumba kwenye fomu hii.

Kusajiliwa kwenye utafiti

Baada ya kusajiliwa, huenda vifaa vya utafiti vikawekwa kwa mtoto wako ili **kuchukua maelezo kuhusu** hali yake (kama mpigo wa moyo, kupumua na kiwango cha joto mwilini), na harakati za kuyumbisha mwili wake. **Vifaa vya utafiti havitoi huduma yoyote ya matibabu kwa mtoto wako na wala havisababishi athari au madhara yoyote kwa mtoto wako. Wahudumu wa afya wa hospitali ndio watatoa huduma za matibabu kwa mtoto wako na wala sio wafanyikazi kwenye utafiti huu, ijapokuwa muuguzi wa utafiti anaweza kusaidia kutoa huduma za kawaida kama hali itabidi wahudumu wa afya hospitalini wakimuhitaji.** Vifaa vya utafiti huenda vikabakia kwa mtoto wako kwa muda wote atakaokuwa bado amelazwa hospitalini lakini havitasumbua utoaji wa huduma za kawaida kwake. Huenda tukachukua picha ama picha za video kwa mtoto wako **lakini picha hazitchukuwa sura yake**. Tutadhibitisha nawe kabla ya kuchukua picha ama video hizi. Mtoto wako akiwa kwenye utafiti huu, **bado ataendelea kupata huduma kutoka kwa wahudumu wa afya hospitalini bila kutatizwa.** Kwa mfano, Daktari **wa hospitali** akiona mtoto wako anahitaji kupewa dawa au kama anahitaji mkumbatio wa mama yake (Mfano wa Kangaroo anavyomtunza mtoto wake), mtoto wako ataweza kupata huduma hizi hata kama yuko kwenye utafiti.

For peer review only - http://bmjopen.bmj.com/site/about/guidelines.xhtml

Ukiamua mtoto wako ajiunge na utafiti huu, tutakuomba ufanye mambo yafuatayo wakati tunapomsajili kwenye utafiti:

- Uweke sahihi yako au alama ya kidole gumba kwenye fomu hii baada ya kuisoma (au baada ya kusomewa), kama unauelewa utafiti wenyewe, na kama umepata nafasi ya kuuliza maswali uliyokuwa nayo kuhusu utafiti huu
- Utoe maelezo kwa muhudumu wa utafiti kuhusu familia yako (Kwa mfano hali ya elimu na mapato ya kiuchumi)
- Utoe maelezo kwa muhudumu wa utafiti uja uzito wa mtoto uliye naye ulichukuwa muda gani.
- Utoe maelezo kwa muhudumu wa utafiti mtoto uliyenaye ulijifungua kwa njia gani (Ulizaa kwa njia ya kawaida ama kwa njia ya upasuaji)
- Utoe maelezo kwa muhudumu wa utafiti kama mtoto wako ni mapacha ama uliwazaa watatu kwa hiyo mimba moja.
- Utoe maelezo kwa muhudumu wa utafiti kuhusu matatizo yoyote ya kiafya ambayo mtoto wako amekuwa nayo.
- Muhudumu wa utafiti atachukua na kurekodi uzani wa mtoto wako.
- Hali ya kiafya ya mtoto wako itafuatiliziwa kutumia vifaa vya utafiti, na hivi vifaa vya utafiti huenda vikaekelewa kwa mtoto wako.
- Mambo yote (kama vile kupewa dawa au mfano wa kangaroo anavyomtunza mtoto wake) yatakayofanyiwa mtoto wako hospitalini yatanakiliwa.

Mbadala/Badala ya kushiriki kwenye utafiti

Uko na chaguo/uamuzi wa kutoshiriki kwenye utafiti huu. Hakutakuwa na matokeo yoyote mabaya kwako au kwa mtoto wako ukiamua/ukichagua kutoshiriki kwenye utafiti huu. Hata ukiamua kutoshiriki mtoto wako **bado ataendelea** kupata huduma za matibabu zenye kiwango cha kawaida. **kutoka hapa hospitali – wanaotoa huduma za matibabu.**

Kwa nini Daktari anaweza kumtoa mtoto wangu kwenye utafiti huu mapema?
Daktari huenda akamtoa mtoto wako kwenye utafiti mapema ikiwa:

- Kama utafiti utasimamishwa ni mdhamini, mfadhili, kamati inayosimamia maadili ya utafiti ama bodi yoyote inayodhibiti mambo ya utafiti.
- Kama mtoto wako ataekelewa mitambo ama kifaa chochote cha kumsaidia kupumua
- Kama mtoto wako atatolewa hospitalini.
- Ama sababu zingine ambazo zinaweza kukuzuwia wewe/mtoto wako kumaliza utafiti kikamilifu.

Kuna Athari Gani Zinazotokana na Kushiriki Kwenye Utafiti Huu?

- Kujibu maswali kunaweza kukufanya usijisikie huru ama kuwa na wasiwasi. Uko huru kutojibu maswali yoyote ambayo hutaki kuyajibu.

For peer review only - http://bmjopen.bmj.com/site/about/guidelines.xhtml

- Walezi wa watoto wanaweza kushiriki kwenye utafiti huu kutokana na shinikizo la kutaka watoto wao wapate huduma za afya ndani ya utafiti, ambayo inaweza kuonekana kama huduma ya kiwango cha hali ya juu sana kuliko kiwango cha matibabu cha kadri ya kawaida.
- Kushiriki kwako kwenye utafiti huu huenda kukaathiri matibabu/uangalizi wa mtoto mchanga aliyelazwa hospitalini, kama taratibu za utafiti zitapewa kipau mbele. Wafanyikazi kwenye utafiti huu watahakikisha hali hii haitukei, na watoto wengine huenda wasisajiliwe kwenye utafiti huu kama wafanyikazi kwenye utafiti huu wataona kwamba kuwajumuisha kwenye utafiti huenda kukacheleweshsa au kutatiza kupata huduma za matibabu kwa njia moja au nyengine.
- Kuweka na kupachika vifaa vya utafiti kwa mtoto mchanga huenda kukasababisha dhiki ama usumbufu kwa muda kidogo. Kupachika vifaa vya utafiti kwa mtoto (Kwa mfano kupachika vifa kwa mwili ama wakati unaotumika kupachika vifaa vya utafiti kwa mwili wa mtoto) pia huenda kukachelewesha mtoto kupata matibabu. Hata hivyo wafanyi kazi kwenye utafiti huu watahakikisha hali kama hii haitokei, na watoto wengine huenda hawatasajiliwa kwenye utafiti ikiwa wafanyi kazi kwenye utafiti wataamini kwamba kujumuishwa kwao kwenye utafiti huenda kukachelewesha ama kutatiza wao kupata matibabu kwa wakati unaofaa.

Je, kuna faida zozote zinazotokana na kushiriki kwenye utafiti huu?

Mtoto wako hatapata manufaa mengine yoyote ya ziada kwa kujiunga na utafiti huu. Utafit huu unafanywa ili kuelewa jinsi ya kubuni/kuendeleza Teknolojia zenye usalama, ufanisi na ambazo haziingiliani za kuangalia afya za watoto wachanga ili kutumika mahali kama Aga Khan University Hospital na Hospitali ya kujifungulia akina mama ya Pumwani. Tunatumaini kwamba hapo siku za usoni Teknolojia hii itasaidia watoto kama huyu wako.

Na je, ni vipi kuhusu usiri?

Tutafanya kila tuwezalo kuhakikisha maelezo yako na ya mtoto wako ya kibinafsi yamewekwa siri. Hata hivyo hatuwezi kukuhakikishia kwamba tutaweka siri ya maelezo yote kwa asilimia kwa mia. Na kama utafiti huu utachapishwa, jina lako/la mtoto wako halitatumka wala hamtatambulishwa. Picha au video zote za mtoto wako hazitakuwa/hazitachukuwa sura yake.

Ili kuhakikisha ikiwa utafiti huu unafanyika vizuri, rekodi/kumbukumbu zako zinaweza kuangaliwa/kupitiwa ni:

- Wafanyikazi kwenye utafiti huu na wafuatiliaji wa utafiti huu.
- Kamati inayosimamia maadili mema ya utafiti ama Institutional Review Board (IRB)
- Taasisi ya Save the Children
- Chuo kikuu cha British Columbia
- Wenye kutengeneza vifaa (EarlySense and Sonica)

Reckodi zote za utafiti za mtoto wako zitawekwa kwenye kliniki/Hospitali kwa muda usiopungua miaka mitano baada ya kumalizika kwa utafiti ama kwa muda unaohitajika kisheria hapa inchini.

For peer review only - http://bmjopen.bmj.com/site/about/guidelines.xhtml

Pia unaweza kuona rekodi za mtoto wako ukizihitaji. Na ukiamua kujitoa kwenye uafiti, maelezo yaliyopatikana kutoka kwa mtoto wako bado yatatumika kwenye utafiti huu.

Je nitatozwa ada/garama ya kiasi gani kwa kushiriki kwenye utafiti huu?
Hautalipa ada au garama zozote kwa kushiriki kwenye utafiti huu.

Je, nitapata malipo yoyote kwa kushiriki kwenye utafiti huu?
Hautapata malipo yoyote kwa kushiriki kwenye utafiti huu.

Ni nini kitatokea kama mtoto wangu atajeruhiwa?
Hakuna uwezekano wa mtoto wako kujeruhiwa kutokana na kujiunga na utafiti huu. Na kama mtoto wako atajeruhiwa kutokana na kujiunga na utafiti huu, mtoto wako atapewa matibabu haraka sana.
Unaweza kuwasiliana na Daktari William Macharia kupitia kupitia nambari ya simu 254-20-3661017 na atakuambia ni wapi mtoto wako ataenda kupata matibabu.

Je, haki za mtoto wangu kama mshiriki kwenye utafiti huu ni zipi?
Uamuzi wa iwapo mtoto wako atashiriki kwenye utafiti huu ni wako kabisa. Ni chaguo lako. Unaweza kuamua mtoto wako akome/awache kufanyiwa taratibu za utafiti wakati wowote – hautalipizwa faini yoyote ama kukosa manufaa/faida ambazo wewe na mtoto wako ni haki yenu. Wewe na mtoto wako mutahudumiwa kiusawa bila kujali uamuzi wako uliochukua. Ukiamua mtoto wako asijiunge na utafiti huu, wewe na mtoto wako hamtakosa faida zozote za huduma ambazo kwa kawaida huwa munapata katika kliniki hii na hautatozwa faini yoyote ama kukosa manufaa/faida zozote ambazo ni haki yenu wewe na mtoto wako.

Tutakupatia maelezo mapya kuhusu utafiti huu ama tafiti zingine kama hizi ambayo huenda yakaathiri afya ya mtoto wako, maslahi yake ama kukubali kwako kuendelea kushiriki kwenye utafiti huu. Kama unataka majibu ya utafiti huu, mjulishe mfanyi kazi katika utafiti huu kwamba utayahitaji.

Maelezo ya utafiti huu yanapatikana kwenye mtandao ufuatao http://www.ClinicalTrials.gov , kama inavyohitajika kisheria inchini Marekani. Mtandao huu hautakuwa na maelezo yanayokutambulisha wewe na mtoto wako. Zaidi sana, mtandao huu utajumuisha matokeo ya utafiti kwa ufupi/mukhtasari. Unaweza kuutafuta mtandao huu wakati wowote.

Utafiti huu uliangaliwa na kupitishwa ni bodi ya Western Institution Review Board iliyoko mjini Washington, Marekani; Kamati ya kuangalia maadili meme ya utafiti ya Chuo Kikuuu cha Aga Khan; Bodi ya kuangalia ubora na usalama wa Madawa na Sumu; Bodi ya Kitaifa ya Sayansi na Teknolojia – Kamati ya Kitaifa ya kuangalia Maadili ya Kibayolojia. Hata hivyo kupitishwa kwa utafiti huu haimaanishi kwamba utafiti uko salama au haimaanishi kwamba Kamati hizi zilipitisha mtoto wako ajiunge na utafiti huu.

For peer review only - http://bmjopen.bmj.com/site/about/guidelines.xhtml

Je, nitafanya nini kama nina matatizo au maswali?

Kama uko na maswali, shaka au malalamiko kuhusu utafiti huu, haki za mtoto kama mshiriki kwenye utafiti, au kama mtoto wako ana jeraha lililotokana na utafiti, unatakiwa kuwasiliana na:

- Daktari William Macharia, Hospitali ya Chuo Kikuu cha Aga Khan , 3rd Parklands Avenue. Tel. 254-(0)203661017. Barua Pepe: william.macharia@aku.edu

SAHIHI

Nimesoma (au nimesomewa na nikapewa maelezo) fomu ya maelezo na makubaliano ya kushiriki kwenye utafiti na maswali yangu yote yamejibiwa. Nimemjulisha mfanyi kazi wa utafiti huu kwamba nimeelewa na ninakubali mtoto wangu ashiriki kwenye utafiti huu. Sahihi yangu au alama yangu ya dole gumba hapo chini yathibitisha kukubali kushiriki kwangu kwa hiari.

____

Jina la Mshiriki
(Mzazi/Mlezi anayetambulika kisheria aandike jina la mtoto atakayeshiriki kwenye utafiti)

____________________	____________________
Jina la Mzazi/Mlezi anayetambulika Kisheria (Andika Jina)	Sahihi ya Mzazi/Mlezi na tarehe
____________________	____________________
Jina la mfanyi kazi kwenye utafiti Anayepeana maelezo/maxungumzo (Andika Jina)	Sahihi ya mfanyi kazi kwenye utafiti na tarehe
____________________	____________________
Jina la shahidi (Andika Jina) (Kama inavyotakikana, ikiwa mzazi/mlezi Anayetambulika kisheria hajui kusoma)	Sahihi ya Shahidi na Tarehe

ETNA Protocol version 1.1, 18 June 2019

For peer review only - http://bmjopen.bmj.com/site/about/guidelines.xhtml

For peer review only

Informed Consent Form for Enrollment

Evaluation of Technologies for Neonates in Africa (ETNA) Study
Healthcare Provider/Administrator Social Sciences Sub-Study
Version 1.0, 14 December 2018

Protocol title: Evaluation of Technologies for Neonates in Africa (ETNA) project

Sponsor: Save the Children Federation, Inc., United States

LOCAL PRINCIPAL INVESTIGATOR AND STUDY CONTACT:
Dr. William Macharia
Aga Khan University, 3rd Parklands Avenue,
P O Box 30270, Nairobi
Contact phone: 254-20-3661017

Part 1: Information Sheet

Introduction

You are being asked to take part in this study because you are a healthcare provider or administrator involved in the Evaluation of Technologies for Neonates in Africa (ETNA) study. The person in charge of this study at this site is Dr. William Macharia. The enrollment process includes a set of questions about the use of continuous physiologic monitoring devices used to monitor the vital signs of neonates.

This is a consent form. It gives you information about the study questions and what you have to do to be in the study. You are free to ask questions about the study at any time. If you agree to take part in this study, you will be asked to sign this consent form. You will be given a copy of this form to keep. Another copy will stay with the study records.

Your participation is voluntary and you may refuse to join or withdraw your consent at any time without any penalty. Before you decide, you can talk to anyone you feel comfortable with about the research. If there is anything that you do not understand, please ask at any time.

Why is this Study Being Done?

The goal of the ETNA study is to optimize and test two continuous physiologic monitoring devices, the EarlySense system and the ANNE system, against reference standard devices. In Kenya, there is a great need for safe and efficient newborn care technologies that are non-invasive, low-cost, and can operate without the constant oversight of a healthcare provider. While this project aims to adapt and test devices that can reliably monitor a newborn's vital signs (such as heart rate, breathing, and temperature) and health status continuously, it is also important for us to understand what healthcare providers and administrators think about the use of these continuous monitoring devices in a hospital setting. In the ETNA sub-study, we aim to learn more

For peer review only - http://bmjopen.bmj.com/site/about/guidelines.xhtml

about healthcare providers' and administrators' experience with and perceptions of these continuous physiologic monitoring devices.

The Bill & Melinda Gates Foundation is providing funds for this study to take place. All healthcare providers and administrators involved in the use of continuous physiologic monitoring devices during the ETNA study may be asked to take part in this study. This study is one visit only.

<u>What Do We Expect to Learn From This Study?</u>

- Do healthcare providers and administrators think that the use of the EarlySense system and the ANNE system is feasible in a hospital or clinical setting (i.e., could these devices be easily used to assist with care of neonates in the hospital)?
- What do healthcare providers and administrators like and dislike about these continuous physiologic monitoring devices?
- How easy is it for healthcare providers to learn how to use these continuous physiologic monitoring devices?
- What problems do healthcare providers encounter when using these continuous physiologic monitoring devices?

<u>What Do I Have To Do If I Take Part in the Study?</u>

If you agree to take part in the study, you will participate in one study visit. This study visit will take about 30 minutes. After signing this consent form, you will be asked questions about your experience with and perception of each of the continuous physiologic monitoring devices. An audio recording may be made of this interview. No study activities will begin before they have been fully explained to you, you have let us know that you understand the study and you have signed this form.

Alternative to study participation

You have the option to not participate in this study. There will be no negative consequences to you or your employment if you choose not to participate in this study.

<u>What Are the Risks of Being in the Study?</u>

Answering questions may make you feel nervous or uncomfortable. You are free to skip any questions that you do not wish to answer.

<u>Are There Benefits To Taking Part In This Study?</u>

There are no direct benefits to you for taking part in this study. However, the study will help healthcare researchers to learn more about continuous physiologic monitoring devices and how they might be improved for use in neonates in hospital settings. This may benefit the clinical outcomes of babies in the future.

For peer review only - http://bmjopen.bmj.com/site/about/guidelines.xhtml

What About Confidentiality?

Efforts will be made to keep your personal information private. If this study is published, your name will not be used and you will not be personally identified.

In order to make sure the study is being done properly, your records may be reviewed by:

- Study staff and monitors
- Ethics Committee and/ or Institutional Review Board (IRB)
- Save the Children, US

Your study records will be kept at the clinic/hospital for at least five years after the study is completed.

What Are The Costs To Me?

There is no cost to you for participating in this study.

What Are My Rights as a Research Participant?

Taking part in research is completely up to you. It's your choice. You may choose to stop at any time. If you choose not to be in the study, there will be no penalty to you or to your employment.

We will tell you about new information from this or other similar studies that may affect your willingness to stay in this study. If you want the results of the study, let the study staff know that you would like them.

The research study was reviewed and approved by the Western Institutional Review Board in Washington, USA; the Aga Khan University Research Ethics Committee; the Pharmacy and Poisons Board; the National Council for Science and Technology - National Bioethics Committee. This approval does not mean that the study is safe or that the committees approved your participation.

What Do I Do If I have Problems or Questions?

For questions, concerns or complaints about the study, you should contact Dr. William Macharia at 7th Floor, East Tower Block, Aga Khan University (contact phone: 254-20-3661017). We do not anticipate that you will be injured due to participation in this study.

For questions, concerns or complaints about the study or questions about your rights as a research participant, contact the Aga Khan University Research Ethics Committee.

For peer review only - http://bmjopen.bmj.com/site/about/guidelines.xhtml

Part 2: Certificate of Consent for Study Participation

SIGNATURE

I have read the informed consent and all of my questions have been answered. I have let the study staff know that I understand and I agree to take part in this study. My signature below documents my consent.

Participant Name (print name)	Participant Signature	Date
Study Staff Name (print name)	Study Staff Signature	Date

For peer review only

For peer review only - http://bmjopen.bmj.com/site/about/guidelines.xhtml

BMJ Open

Evaluation of noninvasive continuous physiological monitoring devices for neonates in Nairobi, Kenya: A research protocol

Journal:	*BMJ Open*
Manuscript ID	bmjopen-2019-035184.R1
Article Type:	Protocol
Date Submitted by the Author:	13-Dec-2019
Complete List of Authors:	ginsburg, amy sarah; Save the Children Federation Inc<br>Nkwopara, Evangelyn; Save the Children Federation Inc<br>Macharia, William; The Aga Khan University, Paediatrics<br>Ochieng, Roseline; Aga Khan University - Kenya, Faculty of Health Sciences<br>Waiyego, Mary ; Pumwani Maternity Hospital<br>Zhou, Guohai; Brigham and Women's Hospital<br>Karasik, Roman; EarlySense<br>Xu, Shuai; Sibel Inc.; Northwestern University<br>Ansermino, J. Mark; The University of British Columbia
**Primary Subject Heading**:	Paediatrics
Secondary Subject Heading:	Paediatrics
Keywords:	NEONATOLOGY, Physiology < BASIC SCIENCES, Quality in health care < HEALTH SERVICES ADMINISTRATION & MANAGEMENT

SCHOLARONE™
Manuscripts

I, the Submitting Author has the right to grant and does grant on behalf of all authors of the Work (as defined in the below author licence), an exclusive licence and/or a non-exclusive licence for contributions from authors who are: i) UK Crown employees; ii) where BMJ has agreed a CC-BY licence shall apply, and/or iii) in accordance with the terms applicable for US Federal Government officers or employees acting as part of their official duties; on a worldwide, perpetual, irrevocable, royalty-free basis to BMJ Publishing Group Ltd ("BMJ") its licensees and where the relevant Journal is co-owned by BMJ to the co-owners of the Journal, to publish the Work in this journal and any other BMJ products and to exploit all rights, as set out in our licence.

The Submitting Author accepts and understands that any supply made under these terms is made by BMJ to the Submitting Author unless you are acting as an employee on behalf of your employer or a postgraduate student of an affiliated institution which is paying any applicable article publishing charge ("APC") for Open Access articles. Where the Submitting Author wishes to make the Work available on an Open Access basis (and intends to pay the relevant APC), the terms of reuse of such Open Access shall be governed by a Creative Commons licence – details of these licences and which Creative Commons licence will apply to this Work are set out in our licence referred to above.

Other than as permitted in any relevant BMJ Author's Self Archiving Policies, I confirm this Work has not been accepted for publication elsewhere, is not being considered for publication elsewhere and does not duplicate material already published. I confirm all authors consent to publication of this Work and authorise the granting of this licence.

For peer review only

Title: Evaluation of noninvasive continuous physiological monitoring devices for neonates in Nairobi, Kenya: A research protocol

Authors: Amy Sarah Ginsburg,[1]* Evangelyn Nkwopara,[2] William Macharia,[3] Roseline Ochieng,[3] Mary Waiyego,[4] Guohai Zhou,[5] Roman Karasik,[6] Shuai Xu,[7,8] and J. Mark Ansermino[9]

[1] University of Washington, Seattle, Washington, USA

[2] Children's Healthcare of Atlanta, Atlanta, Georgia, USA

[3] Aga Khan University, Nairobi, Kenya

[4] Pumwani Maternity Hospital, Nairobi, Kenya

[5] Brigham and Women's Hospital, Harvard Medical School, Boston, Massachusetts, USA

[6] EarlySense Ltd., Ramat-Gan, Israel

[7] Sibel Inc., Evanston, Illinois, USA

[8] Northwestern University, Evanston, Illinois, USA

[9] The University of British Columbia, Vancouver, Canada

For peer review only - http://bmjopen.bmj.com/site/about/guidelines.xhtml

* Corresponding author:

Mailing address: University of Washington Clinical Trial Center

Building 29, Suite 250, 6200 NE 74th Street

Seattle, WA, 98115, USA

Phone: (206)616-8014

Email: messageforamy@gmail.com

For peer review only

For peer review only - http://bmjopen.bmj.com/site/about/guidelines.xhtml

ABSTRACT

Introduction: Continuous physiological monitoring devices are often not available for monitoring high-risk neonates in low-resource settings. Easy-to-use, noninvasive, multiparameter, continuous physiological monitoring devices could be instrumental in providing appropriate care and improving outcomes for high-risk neonates in these low-resource settings.

Methods and Analysis: The purpose of this prospective, observational, facility-based evaluation is to provide evidence to establish whether two existing noninvasive, multiparameter, continuous physiological monitoring devices developed by device developers, EarlySense and Sibel, can accurately and reliably measure vital signs in neonates (when compared to verified reference devices). We will also assess the feasibility, usability and acceptability of these devices for use in neonates in low-resource settings in Africa. Up to 500 neonates are enrolled in two phases: 1) a verification and accuracy evaluation phase at Aga Khan University - Nairobi; and 2) a clinical feasibility phase at Pumwani Maternity Hospital in Nairobi, Kenya. Both quantitative and qualitative data are collected and analyzed. Agreement between the investigational and reference devices is determined using *a priori*-defined accuracy thresholds.

Ethics and Dissemination: This trial was approved by the Aga Khan University Nairobi Research Ethics Committee and the Western Institutional Review Board. We plan to disseminate research results in peer-reviewed journals and international conferences.

ClinicalTrials.gov NCT03920761

For peer review only - http://bmjopen.bmj.com/site/about/guidelines.xhtml

For peer review only

49 **Keywords:** neonates, continuous physiological monitoring devices

For peer review only

For peer review only - http://bmjopen.bmj.com/site/about/guidelines.xhtml

STRENGTHS AND LIMITATIONS OF THIS STUDY

- This research consists of two phases, a verification and accuracy evaluation phase and a clinical feasibility phase, and evaluation of two novel, investigational, noninvasive, multiparameter, continuous physiological monitoring devices.
- A verification of the reference devices is undertaken prior to initiating the accuracy evaluation of the investigational devices to ensure the reference devices are robustly functional and to confirm their within subject repeatability and accuracy compare to standard clinical measurements for the relevant parameters of interest.
- Reliability information gathered from the reference devices is utilized to determine specific *a priori* Go/No Go criteria for each parameter and each investigational device.
- As with all measurements, there is uncertainty inherent in the measurements from the reference devices.
- Inability to control for the characteristics and conditions of the participating neonates and to standardize the environment and context are both strengths and limitations to interpreting the results.

For peer review only - http://bmjopen.bmj.com/site/about/guidelines.xhtml

INTRODUCTION

In 2017 globally, 47% of all deaths in children under 5 years of age occurred within the first 28 days of life, which translates to a neonatal mortality rate of 18 deaths per 1000 live births or 2.5 million newborn deaths.[1] Sub-Saharan Africa bears the greatest burden of neonatal mortality with an estimated 1 million newborn deaths in 2017. Further efforts, especially in African countries, are needed to push progress towards achieving the Sustainable Development Goal (SDG) target of reducing global neonatal mortality to 12 deaths per 1000 live births by 2030.[2] Without accelerated improvements, it is projected that 1.8 million neonates will die in 2030.[3] Innovations in neonatal care, particularly technologies that allow for early detection and intervention for major morbidities, hold great promise in helping to reduce current and projected neonatal mortality rates.

Multiparameter continuous physiological monitoring devices could be instrumental in identifying neonates at risk. We can then direct care provided for a neonate through automatic interpretations of vital signs that help identify critical events and determine if treatment is sufficient or insufficient, ultimately improving newborn outcomes.[4,5] These devices would be most useful in low-resource settings where the need for such technologies is greatest. While continuous physiological monitoring is standard of care in high-resource settings for those who require it, the devices are expensive and require specialized training to operate, making them unsuitable for application in low-resource settings. To address these barriers, it is necessary to explore how these technologies can be adapted and/or optimized for use in low-resource settings. Ideally, the devices should be low cost, operator-independent, noninvasive and highly efficient in diagnostic performance and operator workload. This requires development of a robust testing platform that appropriately mimics

For peer review only

For peer review only - http://bmjopen.bmj.com/site/about/guidelines.xhtml

conditions common in African newborn or neonatal intensive care units that would allow these type of technologies to be evaluated for feasibility and performance.

The Evaluation of Technologies for Neonates in Africa (ETNA) project was conceived with the goal of advancing and supporting development, as well as evaluation, of select devices for use in neonates in low-resource settings. By establishing a testing platform in an African site, and working collaboratively with partners with expertise in device development and evaluation and neonatal and child health, the project seeks to boost development and optimization of promising newborn care devices that could be applied in low-resource settings in Africa. We acknowledge the many challenges involved in implementing such devices in low-resource settings (e.g., electricity and internet access, behavior change communication, etc.), and the need to consider these challenges carefully prior to introduction. The purpose of this initial research is to produce evidence regarding the performance of two existing noninvasive, multiparameter, continuous physiological monitoring devices developed by device developers, EarlySense and Sibel, to accurately and reliably measure vital signs in neonates (when compared to verified reference devices) and to assess the feasibility, usability and acceptability of these devices for use in neonates in a low-resource setting in Africa.

METHODS AND ANALYSIS

Study design and setting

The primary objectives of this prospective, observational, facility-based research are: 1) to assess agreement between repeat observations by the investigational device and the

For peer review only - http://bmjopen.bmj.com/site/about/guidelines.xhtml

reference device for each relevant measurement parameter of interest based on *a priori*-determined accuracy threshold among neonates; 2) to compare clinical event detection performance between the investigational device and the reference device; and 3) to determine whether the investigational device is feasible, usable and acceptable to hospital administrators, healthcare providers and caregivers of neonates. Secondary objectives include: 1) assessing diagnostic performance for each relevant measurement parameter of interest based on sensitivity, specificity, positive predictive value, and negative predictive value compared to the reference device; 2) determining the downtime performance of the investigational device; 3) determining the alarm rate (events/hour) and the number of true/false alarms of the investigational device compared to the reference device; 4) determining the delay time between the investigational device and the reference device in true events; and 5) determining the number of adverse device effects (ADEs) and serious adverse events (SAEs) during use of the investigational device.

Beginning in June 2019 and anticipated to last approximately 18 months in Nairobi, Kenya, this research consists of two phases: 1) a verification and accuracy evaluation phase conducted at Aga Khan University – Nairobi (AKU-N), a private, not-for-profit university teaching hospital with a neonatal intensive care and high dependency units; and 2) a clinical feasibility phase conducted at Pumwani Maternity Hospital (PMH), the largest referral maternity hospital in sub-Saharan Africa with no neonatal intensive care or high dependency units.

Study participants

Up to 500 neonates, corrected age of <u><</u>28 days admitted for routine observation and care at AKU-N and PMH are recruited by trained study staff during routine intake and screening procedures. To avoid potential selection bias, neonates are screened for enrollment in a sequential manner, as much as possible. Trained study staff assess the neonate for all

For peer review only - http://bmjopen.bmj.com/site/about/guidelines.xhtml

inclusion and exclusion criteria (Table 1). Final eligibility determination is dependent on the results of the medical history, clinical examination, appropriate understanding of the study by the caregiver, and completion of the written informed consent process. A neonate may be enrolled to the study more than once as long as they meet the eligibility criteria and the caregiver(s) is willing to have the neonate participate.

For the feasibility, usability and acceptability assessment, hospital administrators and study healthcare providers are enrolled if they are 18 years or older, involved in or aware of the ETNA study, and have provided written informed consent. Caregivers may be enrolled if they are 18 years or older, have a neonate enrolled in the study, and are willing to participate in a 30-minute in-depth interview as well as direct observation while their neonate is on or attached to the investigational device(s).

Investigational devices

Developed by Israeli-based EarlySense since 2009, the Insight system, released in 2016, is a contact-free monitoring system comprised of a small piezoelectric sensor pad that can be placed under the patient's mattress, and is designed to measure and record a patient's heart rate (HR), respiratory rate (RR), motion, and sleep status. Information from the sensor pad, in combination with EarlySense's artificial intelligence analytics, is transmitted to a monitor to provide alert indications and vital sign trends to healthcare providers so that they can monitor changes in a patient's condition. Currently in use in hospitals, rehabilitative centers, and nursing homes to measure vital signs in adults and children above 10 kg, the device is modified for use in neonates as part of this study. The adult device received regulatory approval from the United States Federal Drug Administration (FDA) and has a Conformité Européene (CE) mark for continuous and contactless measurement of HR,

For peer review only - http://bmjopen.bmj.com/site/about/guidelines.xhtml

RR and motion. No adverse events (AEs) related to the system have been reported during 10 years of monitoring.

Developed in 2019, the advanced neonatal epidermal (ANNE) system from Sibel, a technology company spun out from the Center of Bio-Integrated Electronics at Northwestern University in the United States, is a system of two time-linked soft and flexible sensors designed to measure and monitor vital signs including HR, RR, oxygen saturation (SpO_2), and skin temperature in neonates. The chest sensor couples to the skin via a hypo-allergenic, biocompatible hydrogel adhesive optimized for reduced peel force upon removal, and the limb unit couples via a latex-free soft fabric wrap adaptable to a range of foot sizes and anatomies. Information from the sensors are wirelessly transmitted to a monitor or mobile device via encrypted Bluetooth™ for real-time streaming from a customized mobile software application as well as onboard memory storage on the sensors themselves. The device has been validated in more than 50 neonates in a neonatal care unit without AEs.

Reference devices

We are employing the Masimo Rad-97™ and the Spengler Tempo Easy Bleu devices as our reference devices for this study. The Masimo Rad-97™ provides continuous physiological monitoring of HR, RR, SpO2, and capnography. The Spengler Tempo Easy Bleu non-contact infrared thermometer predicts core body temperature from the temporal artery temperature.

Study procedures

Following completion of screening for eligibility, a study comprehension checklist, and written informed consent, study staff perform procedures (Appendix I: Schedule of study

For peer review only

For peer review only - http://bmjopen.bmj.com/site/about/guidelines.xhtml

procedures and evaluations) according to the most recently approved version of the protocol (current version 1.1, June 18, 2019). Enrolled neonates are assigned a participant identification number; information is collected on socio-demographic characteristics, current clinical status, medical history, medications; and a physical examination is performed.

Prior to initiating the accuracy evaluation of each investigational device, verification of the reference devices, Masimo Rad-97™ and Tempo Easy Bleu, is undertaken at AKU-N to ensure they are robustly functional and to confirm their within subject repeatability and accuracy compared to standard clinical measurements (e.g., manual, bedside electrocardiography) for the relevant parameters of interest. Neonates enrolled during reference device verification continue to receive local standard of care while being observed intermittently for vital signs collection for a minimum of 1 hour using the Masimo Rad-97™ and intermittent measurements with the Tempo Easy Bleu. Observations may include video recordings of the neonate and the Masimo Rad-97™reference device monitor for later review to facilitate manual count observations. The reference device measurements will be compared to manual measurements, clinical monitor observations, and video-assisted observations. Reliability information gathered from the reference devices is utilized to determine specific Go/No Go criteria for each parameter and each investigational device. Further evaluation of each investigational device only proceeds should these criteria be met.

Enrollment in the accuracy evaluation of the investigational devices, EarlySense Insight system and Sibel ANNE system, is initiated at AKU-N to formally assess their accuracy compared to the verified reference device using repeated observations. Enrolled neonates continue to receive local standard of care while having vital signs collected from the

For peer review only - http://bmjopen.bmj.com/site/about/guidelines.xhtml

reference device as well as one or both of the investigational devices. Placement of the investigational and reference devices is done in a manner so as not to interfere with the neonate's clinical care. Observations are collected for a minimum of 1 hour and potentially for the entire duration of their stay in the hospital. Observations may consist of videotaping and/or taking photos of the neonate during the observation period after obtaining informed consent from the caregiver. Any photos or videos takes are identified by patient identification number only and stored on a secure server until the analyses are completed and destroyed following analyses. During observation, clinical status and any activities are updated and recorded including type and duration of care activities (e.g., feeding, diaper changes, bathing, kangaroo mother care, etc.), clinical procedures, interventions, therapies, laboratory tests, medications, environmental features and exposures during hospitalization. The device placement, output, and signal quality are also monitored. In addition, the neonates are assessed for any safety issues. Agreement between the investigational and reference devices is determined using *a priori*-defined accuracy thresholds. Thresholds are determined largely based on repeated within and between subject observations during verification of the reference devices. This is complemented by previously published international standards where available, and clinical expert consensus opinion as needed. Two *a priori*-determined thresholds are determined: one lower threshold to allow the device developer to optimize the device for retesting, and a second higher threshold to allow the device to move on to the clinical feasibility phase of testing. A maximum of 5 rounds of testing and retesting are permitted for each investigational device. Each round of testing or retesting consists of using a cohort of 20 neonates. Should the lower threshold not be reached for at least one parameter, no further testing of the investigational device is performed. Thus, information collected during the accuracy evaluation along with the *a*

For peer review only - http://bmjopen.bmj.com/site/about/guidelines.xhtml

priori–determined Go/No Go criteria established during verification of the reference devices define which, if any, of the investigational devices moves forward with additional rounds of testing or into the clinical feasibility phase at PMH.

An investigational device advances to the clinical feasibility phase once the agreement for the measurement parameters of interest exceed the higher accuracy threshold. Enrollment in the clinical feasibility phase of the investigational devices occurs at PMH in up to 120 enrolled neonates who receive local standard of care while being monitored with the reference device(s) and one or both of the investigational devices. Observations are collected for a minimum of 1 hour and involve measurement of vital signs via the investigational and reference devices and monitoring for any critical event (i.e., low or high HR, RR, or temperature or oxygen desaturation and apnea). Agreement between repeated observations from the investigational and reference devices as well as diagnostic performance in clinical event detection is evaluated. Additional performance metrics such as alarm rates, alarm delays and uptime\downtime are compared between the investigational and reference devices. Participation in the study does not interfere with or unnecessarily delay the clinical care of the neonates.

For peer review only

Throughout all phases of the research, the investigational devices are not used to inform clinical care. During the clinical feasibility phase, ETNA site study staff and hospital healthcare providers are blinded to the data collected from the investigational devices to prevent interference with clinical care. The study site investigators are responsible for close safety monitoring of all participating neonates, including assessing for and reporting adverse device effects (e.g., erythema or edema at the investigational or reference device sensor site) and/or serious adverse events (i.e., any adverse device effect resulting in permanent

skin damage). Any adverse device effects or serious adverse events will be treated until resolution or stabilization, and may require removal of devices and withdrawal of the neonate from the study if necessary. If withdrawn by the study team, any enrolled neonate who completes at least one hour of monitoring will be included in the analysis and results.

Qualitative substudy

After written informed consent is received from the study participants, a mixed methods evaluation and data collection through audio-recorded semi-structured in-depth interviews and direct observations are conducted by trained qualitative study staff to assess the feasibility, usability, and acceptability of the investigational devices for monitoring of neonates in an African-setting. Questions around technology use, experience with continuous monitoring devices, and specific to each investigational and reference device will be asked and their use observed. All hospital administrators and study healthcare providers may be involved in this portion of the study. Caregivers with a neonate enrolled in the study may also be asked if they would like to participate in the qualitative portion of the study.

Sample size

A total of up to 500 neonates are enrolled. For the verification of the reference devices at AKU-N, up to 30 neonates are enrolled. Once this initial testing and data collection of the reference devices are complete, for the accuracy evaluation phase at AKU-N, up to 120 neonates per investigational device are enrolled. Sample size estimates for the verification of the reference devices and the accuracy evaluation phase are based on the confidence intervals (CIs) desired for the limits of agreement. Sample sizes of 100-200 typically provide tight CIs. A sample of 20 neonates with 10 replications per neonate per device per round of

For peer review only

For peer review only - http://bmjopen.bmj.com/site/about/guidelines.xhtml

testing provides limits of agreement with 95% CIs +/- 0.24, calculated as 1.96*sqrt(3/(20*10)), times the standard deviation of the paired differences. The paired differences are from the reference device and manual measurements obtained during verification of the reference device, and from the reference device and investigational device measurements obtained during the accuracy evaluation phase. For the clinical feasibility phase at PMH, up to 120 neonates per investigational device are enrolled. The sample sizes for each phase have been selected to maximize the amount of information collected within the confines of the available resources.

For the feasibility, usability, and acceptability assessment, the total sample size includes all hospital administrators and study healthcare providers willing to participate and provide consent as well as up to 30 caregivers willing to participate and provide consent study at each site.

Data collection and quality assurance

Quantitative study data is collected by clinical study staff using designated source documents as well as electronic or paper-based case report forms. Data is stored and managed by a database developed via Research Electronic Data Capture (REDCap), a secure web application. Continuous physiological data and event data are recorded from the investigational and reference devices at least once a second. All electronic data are collected wirelessly or via a wired connection from the investigational and reference devices to a study laptop using custom software applications. Qualitative study data is collected using paper-based forms and audio recordings which are subsequently transcribed for analysis.

For peer review only

For peer review only - http://bmjopen.bmj.com/site/about/guidelines.xhtml

295 Clinical research data, including data collected from the investigational and reference devices, are maintained through a combination of secure electronic data management system and physical files with restricted access to ensure confidentiality. Two distinct study databases are maintained separately: the primary study database and a database with participating neonate's personally identifiable information. To ensure accuracy and completeness, data is routinely reviewed by the investigators through quality assurance reviews, audits, and evaluation of the study safety and progress. Guideline for Good Clinical Practice (GCP)/ ISO 14155 compliance is followed to ensure accurate, reliable, and consistent data collection.

304 **Data management**

305 Primary data management activities, which include de-identified investigational and reference device data transfer using end-to-end encryption with two-factor authentication, data entry and validation, data cleaning, database quality control, and disaster recovery plans are undertaken at the study site and are overseen by the on-site data manager. Data review and analysis, oversight and preparation of final study database is performed by the investigators in collaboration with the study site. Data are maintained and stored securely in databases hosted at the study site throughout the study and for at least 5 years after study closure. All data management activities are in compliance with International Council on Harmonization (ICH) GCP E6, sponsor organization, and institutional requirements for the protection of children and confidentiality of personal and health information.

315 **Outcomes**

For peer review only

For peer review only - http://bmjopen.bmj.com/site/about/guidelines.xhtml

We hypothesize that the investigational device is accurate and reliable compared to the reference device for each relevant measurement parameter of interest among neonates and is feasible, usable and acceptable for use in neonates in low-resource settings. The primary endpoint and secondary endpoints are detailed in Table 2.

Statistical analyses

Every second of data is automatically graded as optimal, acceptable and unacceptable based on predefined rules for each device and each measurement parameter of interest according to the quality of the data for each measurement parameter of interest. The Masimo Rad-97™ provides a signal quality index that is used to determine data quality for HR and SpO2. A custom algorithm has been produced to determine the capnography signal quality index. Each of the investigational devices also provides a signal quality index. The quality thresholds are determined following verification of the reference devices. All comparisons are performed from observations between two devices (or a single device during verification). At least 10 observations of 60 seconds of optimal quality data in each neonate, at least 5 minutes apart, are randomly selected for each measurement parameter of interest from the full recording. For the clinical feasibility phase, accuracy comparisons use optimal or acceptable data. At least 3 hours of recording to a maximum of 12 hours are used for the performance metrics such as alarm rates, alarm delays and uptime\downtime.

The repeatability of the reference device parameter estimates initially is assessed with the intraclass correlation coefficient (ICC). Additional training or standardization of procedures is performed to ensure at least good repeatability (ICC >0.7). This is followed by measuring agreement between the repeated reference observations and between the manual, clinical monitor and video-assisted methods and the reference observations using the methods

For peer review only - http://bmjopen.bmj.com/site/about/guidelines.xhtml

described by Bland and Altman for replicated observations.[6] The agreement is reported as a mean bias with 95% CIs and 95% limits of agreement. Graphical representation of the data is assessed with agreement plots, Clark error grids, and Polar plots to identify extreme outliers and significant data trends.

In the accuracy evaluation, the root mean square difference (RMSD) and ICC are calculated for each measurement parameter of interest to compare the multiple repeated observations between the investigational and reference devices. The agreement between each investigational device and reference device(s) is then calculated using the methods described by Bland and Altman for replicated observations. The agreement is reported as a mean bias with 95% CIs and 95% limits of agreement. Graphical representation of the data is be assessed with agreement plots, Clark error grids, and Polar plots to identify extreme outliers, impact on clinical decisions, and significant data trends. An *a priori*–defined accuracy margin for agreement is used as a threshold value to allow for decisions regarding proceeding to additional testing.

In the clinical feasibility phase, agreement between each investigational device and reference device(s) is assessed as in the accuracy evaluation phase. Event detection rates, alarm rates, alarm delays and uptime/ downtime are summarized with means, medians, standard deviations and intra-quartile ranges as appropriate. Summaries of sensitivity, specificity, positive predictive values and negative predictive values comparing each measurement parameter of interest in the investigational device(s) to the reference device(s) are produced. Comparisons of binary events are assessed using Cohen’s weighted Kappa and McNemar’s test. The non-inferiority of alarm rates, alarm delays and uptime/ downtime are evaluated based on pre-specified thresholds.

For peer review only

Qualitative data are collected through in-depth interviews and/or semi-structured questionnaires and analyzed to assess feasibility, usability, and acceptability of the investigational devices among hospital administrators and healthcare providers, and acceptability among caregivers of enrolled neonates. Questions that explore familiarity, knowledge, perceptions, attitudes and behaviors regarding the devices are included. The qualitative data is in narrative format and the results are descriptive. The questionnaires are coded and analyzed using a codebook with identified themes, including feasibility of using each investigational device, barriers and facilitators to use, and perceived value. Qualitative data analysis software is used to organize, code, and analyze the qualitative data in an iterative process. The study team starts by identifying an initial set of codes and themes based on the categories from the interview guides. During the coding process, attention is paid to identifying emergent issues and themes that are added to the codebook and included in the analysis. Responses from the interviews are coded and discrepancies discussed and resolved for the final analysis and theme identification.

ETHICS AND DISSEMINATION

Ethical approvals and consent

The study is conducted in accordance with the ICH GCP and the Declaration of Helsinki 2008. The protocol and other relevant study documents study were approved by the Western Institutional Review Board 20191102 (Puyallup, Washington, United States of America), and the Aga Khan University Nairobi Research Ethics Committee 2019/REC-02 (v2)(Nairobi, Kenya). Written informed consent is obtained in the local language by trained study staff

For peer review only - http://bmjopen.bmj.com/site/about/guidelines.xhtml

384 from all eligible neonate's caregivers and for the qualitative substudy, from participating
385 hospital administrators, healthcare providers, and caregivers prior to enrollment. Potential
386 participants will have adequate time to ask questions and a comprehension checklist will be
387 administered to ensure participant understanding.

388 **Possible risks**

389 Caregivers may feel compelled to enroll in the study in order to receive care for their
390 neonate within a research setting, which may be perceived as of a higher quality than the
391 standard of care. In order to minimize the risk of coercion, during the informed consent
392 process, study staff emphasize that the neonate will receive the required medical care
393 whether enrolled in the study or not. Other potential risks to study participation may
394 include those associated with the placement and attachment of the investigational and
395 reference devices, and delayed medical management. Study staff are trained in the
396 appropriate placement of investigational and reference devices' sensors to minimize
397 discomfort to the neonates as well as to avoid interference with any assessment, treatment,
398 or intervention necessary for clinical care. There is a potential risk of skin irritation with the
399 ANNE sensor system and neonates will be closely monitored and treated for any AEs. Study
400 staff are also trained in integrating study procedures with clinical care and to always
401 prioritize clinical care above study procedures. Extreme care is taken to ensure that no
402 necessary treatment is delayed to accommodate study procedures.

403 **Dissemination**

404 We plan to disseminate study results in peer-reviewed journals and international
405 conferences, targeting those involved in the clinical care of neonates in low-resource

For peer review only - http://bmjopen.bmj.com/site/about/guidelines.xhtml

settings as well as those who develop and advise on policies and guidelines in those settings.

The trial is registered with ClinicalTrials.gov (registration number NCT03920761).

Efforts towards rigorous protocol

Dedicated study staff trained in GCP, operation, use and maintenance of the investigational and reference devices, and study-specific procedures follow neonates enrolled in the trial to assure the protocol and standard operating procedures are followed and data are accurately collected. Standardized study-specific training, supervision, and oversight are undertaken to ensure quality, consistency, and harmonized trial procedures and implementation. Regular monitoring is provided by the co-investigators to assess compliance with human subjects and other research regulations and guidelines, adherence to the study protocol and procedures, and quality and accuracy of data collected.

Limitations and bias

Limitations to this study and potential sources of bias include the sampling strategy, the uncertainty inherent in the measurements from the reference devices, the limited standardization of time of day of recording, and the inability to control the conditions and standardize the context. Because there is a large variation in the various ages, weights, sizes, disease states, clinical presentations, interventions received, and conditions of the participating neonates, it is not possible to control for all these variables. Likewise, the environment cannot be controlled, does not allow for complete standardization, and may introduce additional sources of bias. These limitations may also be viewed as strengths.

DECLARATIONS

Authors' contributions ASG, EN, WM and MA designed the study and wrote the protocol.

For peer review only - http://bmjopen.bmj.com/site/about/guidelines.xhtml

RO, MW, GZ, RK, and SX reviewed and provided critical input to the study design and protocol. ASG wrote the first draft of the manuscript, and EN and MA provided additional input. All authors worked collaboratively, reviewed the manuscript, and made the decision to submit the final manuscript for publication.

Acknowledgments We would like to thank Dustin Dunsmuir who wrote the IAP logger application used to collect the high resolution data from the reference device.

Funding This work is supported by grants from the Bill & Melinda Gates Foundation (OPP1203136) and the Save the Children Innovation Council. The authors had final responsibility for the decision to submit this manuscript for publication.

For peer review only

Competing interests RK is employed by EarlySense Ltd. and SX is employed by Sibel Inc. All other authors declare that they have no competing interests.

Ethics approval and consent to participate The study was approved by the Western Institutional Review Board 20191102 (Puyallup, Washington, United States of America) and the Aga Khan University Nairobi Research Ethics Committee2019/REC-02(v2) (Nairobi, Kenya). Written informed consent is obtained by trained study staff from all eligible children’s caregivers prior to enrollment.

Consent for publication Not applicable.

Patient and public involvement Patients and the public were not involved in the design of, recruitment to, or the conduct of the study.

Availability of data and materials Data will be made available on an open access platform after the publication of the main manuscripts. Processes will be developed to facilitate data sharing for scientific utilization in a collaborative manner.

For peer review only - http://bmjopen.bmj.com/site/about/guidelines.xhtml

REFERENCES

1. United Nations Inter-agency Group for Child Mortality Estimation (UN IGME), 'Levels & Trends in Child Mortality: Report 2018, Estimates developed by the United Nations Inter-agency Group for Child Mortality Estimation' United Nations Children's Fund. New York, 2018.

2. Sustainable Development Goals Knowledge Platform. Division for Sustainable Development Goals.[website]. New York, NY: United Nations Headquarters. Date Accessed: September 22, 2019. https://sustainabledevelopment.un.org/sdg3.

3. Hug L, Alexander M, You D, Alkema L, Estimation UNI-aGfCM. National, regional, and global levels and trends in neonatal mortality between 1990 and 2017, with scenario-based projections to 2030: a systematic analysis. *Lancet Glob Health* 2019; **7**(6): e710-e20.

4. Zhu Z, Liu T, Li G, Li T, Inoue Y. Wearable sensor systems for infants. *Sensors (Basel)* 2015; **15**(2): 3721-49.

5. Sahni R. Continuous noninvasive monitoring in the neonatal ICU. *Curr Opin Pediatr* 2017; **29**(2): 141-8.

6. Bland JM, Altman DG. Measuring agreement in method comparison studies. *Stat Methods Med Res* 1999; **8**(2): 135-60.

For peer review only - http://bmjopen.bmj.com/site/about/guidelines.xhtml

TABLES AND FIGURE LEGENDS

Table 1. Eligibility criteria

**Eligibility criteria**	
Inclusion criteria	• Male or female neonate, corrected age of ≤28 days.<br>• Willingness and ability of neonate’s caregiver to provide informed consent and to be available for follow-up for the planned duration of the study.
Exclusion criteria	• Receiving mechanical ventilation or continuous positive airway pressure.<br>• Skin abnormalities in the nasopharynx and/or oropharynx.<br>• Contraindication to application of skin sensors.<br>• Known arrhythmia.<br>• Presence of a congenital abnormality requiring major surgical intervention.<br>• Any medical or psychosocial condition or circumstance that, in the opinion of the investigators, would interfere with the conduct of the study or for which study participation might jeopardize the neonate’s health.

For peer review only

For peer review only - http://bmjopen.bmj.com/site/about/guidelines.xhtml

Table 2. Study endpoints

Primary endpoints

- Agreement of the relevant measurement parameters of interest between the investigational device and the reference device at each observation.
- Agreement of clinical event detection between the investigational device and the reference device at each observation.
- Feasibility, usability and acceptability of the investigational device among hospital administrators and healthcare providers.
- Acceptability of the investigational device among caregivers.

Secondary endpoints

- Diagnostic performance of the investigational device to appropriately identify the following critical events:
    - Low heart rate
    - High heart rate
    - Low respiratory rate
    - High respiratory rate
    - Oxygen desaturation
    - Apnea
    - Low temperature
    - High temperature
- Downtime duration of the investigational device.
- Alarm rate (events/hour and ratio of false positives to missed critical events of the investigational device's alarms compared to the reference device's alarms.

For peer review only

For peer review only - http://bmjopen.bmj.com/site/about/guidelines.xhtml

- Response time of the investigational device's alarms compared to the reference device's alarms for critical events.
- Proportion of neonates with adverse device effects and serious adverse events resulting in skin damage.

For peer review only

For peer review only - http://bmjopen.bmj.com/site/about/guidelines.xhtml

1 **Appendix I: Schedule of study procedures and evaluations**

Activity	Screening	Enrollment	Observation	Discharge
Eligibility assessment	X			
Informed consent and comprehension checklist	X			
Assign participant ID	X			
Demographics	X	X		
Medical history		X	X	X
Maternal pregnancy history		X		
Medications use		X		
Placement of reference and/or investigational device(s)		X	X	
Collection of vital signs		X	X	X
Video tape recording and/or photographs			X	

For peer review only

For peer review only - http://bmjopen.bmj.com/site/about/guidelines.xhtml

Track clinical care and non-study activities			X	X
Safety assessment			X	
End of study questions				X
Removal of investigational and/or reference device(s)				X

2

For peer review only

Evaluation of noninvasive continuous physiological monitoring devices for neonates in Nairobi, Kenya: A research protocol

Journal:	*BMJ Open*
Manuscript ID	bmjopen-2019-035184.R2
Article Type:	Protocol
Date Submitted by the Author:	10-Mar-2020
Complete List of Authors:	ginsburg, amy sarah; Save the Children Federation Inc<br>Nkwopara, Evangelyn; Save the Children Federation Inc<br>Macharia, William; The Aga Khan University, Paediatrics<br>Ochieng, Roseline; Aga Khan University - Kenya, Faculty of Health Sciences<br>Waiyego, Mary ; Pumwani Maternity Hospital<br>Zhou, Guohai; Brigham and Women's Hospital<br>Karasik, Roman; EarlySense<br>Xu, Shuai; Sibel Inc.; Northwestern University<br>Ansermino, J. Mark; The University of British Columbia
**Primary Subject Heading**:	Paediatrics
Secondary Subject Heading:	Paediatrics
Keywords:	NEONATOLOGY, Physiology < BASIC SCIENCES, Quality in health care < HEALTH SERVICES ADMINISTRATION & MANAGEMENT

SCHOLARONE™ Manuscripts

I, the Submitting Author has the right to grant and does grant on behalf of all authors of the Work (as defined in the below author licence), an exclusive licence and/or a non-exclusive licence for contributions from authors who are: i) UK Crown employees; ii) where BMJ has agreed a CC-BY licence shall apply, and/or iii) in accordance with the terms applicable for US Federal Government officers or employees acting as part of their official duties; on a worldwide, perpetual, irrevocable, royalty-free basis to BMJ Publishing Group Ltd ("BMJ") its licensees and where the relevant Journal is co-owned by BMJ to the co-owners of the Journal, to publish the Work in this journal and any other BMJ products and to exploit all rights, as set out in our licence.

The Submitting Author accepts and understands that any supply made under these terms is made by BMJ to the Submitting Author unless you are acting as an employee on behalf of your employer or a postgraduate student of an affiliated institution which is paying any applicable article publishing charge ("APC") for Open Access articles. Where the Submitting Author wishes to make the Work available on an Open Access basis (and intends to pay the relevant APC), the terms of reuse of such Open Access shall be governed by a Creative Commons licence – details of these licences and which Creative Commons licence will apply to this Work are set out in our licence referred to above.

Other than as permitted in any relevant BMJ Author's Self Archiving Policies, I confirm this Work has not been accepted for publication elsewhere, is not being considered for publication elsewhere and does not duplicate material already published. I confirm all authors consent to publication of this Work and authorise the granting of this licence.

For peer review only

Title: Evaluation of noninvasive continuous physiological monitoring devices for neonates in Nairobi, Kenya: A research protocol

Authors: Amy Sarah Ginsburg,[1]* Evangelyn Nkwopara,[2] William Macharia,[3] Roseline Ochieng,[3] Mary Waiyego,[4] Guohai Zhou,[5] Roman Karasik,[6] Shuai Xu,[7,8] and J. Mark Ansermino[9]

[1] University of Washington, Seattle, Washington, USA

[2] Children's Healthcare of Atlanta, Atlanta, Georgia, USA

[3] Aga Khan University, Nairobi, Kenya

[4] Pumwani Maternity Hospital, Nairobi, Kenya

[5] Brigham and Women's Hospital, Harvard Medical School, Boston, Massachusetts, USA

[6] EarlySense Ltd., Ramat-Gan, Israel

[7] Sibel Inc., Evanston, Illinois, USA

[8] Northwestern University, Evanston, Illinois, USA

[9] The University of British Columbia, Vancouver, Canada

For peer review only

For peer review only - http://bmjopen.bmj.com/site/about/guidelines.xhtml

* Corresponding author:

Mailing address: University of Washington Clinical Trial Center

Building 29, Suite 250, 6200 NE 74th Street

Seattle, WA, 98115, USA

Phone: (206)616-8014

Email: messageforamy@gmail.com

For peer review only

For peer review only - http://bmjopen.bmj.com/site/about/guidelines.xhtml

ABSTRACT

Introduction: Continuous physiological monitoring devices are often not available for monitoring high-risk neonates in low-resource settings. Easy-to-use, noninvasive, multiparameter, continuous physiological monitoring devices could be instrumental in providing appropriate care and improving outcomes for high-risk neonates in these low-resource settings.

Methods and Analysis: The purpose of this prospective, observational, facility-based evaluation is to provide evidence to establish whether two existing noninvasive, multiparameter, continuous physiological monitoring devices developed by device developers, EarlySense and Sibel, can accurately and reliably measure vital signs in neonates (when compared to verified reference devices). We will also assess the feasibility, usability and acceptability of these devices for use in neonates in low-resource settings in Africa. Up to 500 neonates are enrolled in two phases: 1) a verification and accuracy evaluation phase at Aga Khan University - Nairobi; and 2) a clinical feasibility phase at Pumwani Maternity Hospital in Nairobi, Kenya. Both quantitative and qualitative data are collected and analyzed. Agreement between the investigational and reference devices is determined using *a priori*-defined accuracy thresholds.

Ethics and Dissemination: This trial was approved by the Aga Khan University Nairobi Research Ethics Committee and the Western Institutional Review Board. We plan to disseminate research results in peer-reviewed journals and international conferences.

ClinicalTrials.gov NCT03920761

For peer review only - http://bmjopen.bmj.com/site/about/guidelines.xhtml

49 **Keywords:** neonates, continuous physiological monitoring devices

For peer review only

For peer review only - http://bmjopen.bmj.com/site/about/guidelines.xhtml

STRENGTHS AND LIMITATIONS OF THIS STUDY

- This research consists of two phases, a verification and accuracy evaluation phase and a clinical feasibility phase, and evaluation of two novel, investigational, noninvasive, multiparameter, continuous physiological monitoring devices.
- A verification of the reference devices is undertaken prior to initiating the accuracy evaluation of the investigational devices to ensure the reference devices are robustly functional and to confirm their within subject repeatability and accuracy compare to standard clinical measurements for the relevant parameters of interest.
- Reliability information gathered from the reference devices is utilized to determine specific *a priori* Go/No Go criteria for each parameter and each investigational device.
- As with all measurements, there is uncertainty inherent in the measurements from the reference devices.
- Inability to control for the characteristics and conditions of the participating neonates and to standardize the environment and context are both strengths and limitations to interpreting the results.

For peer review only - http://bmjopen.bmj.com/site/about/guidelines.xhtml

INTRODUCTION

In 2017 globally, 47% of all deaths in children under 5 years of age occurred within the first 28 days of life, which translates to a neonatal mortality rate of 18 deaths per 1000 live births or 2.5 million newborn deaths.[1] Sub-Saharan Africa bears the greatest burden of neonatal mortality with an estimated 1 million newborn deaths in 2017. Further efforts, especially in African countries, are needed to push progress towards achieving the Sustainable Development Goal (SDG) target of reducing global neonatal mortality to 12 deaths per 1000 live births by 2030.[2] Without accelerated improvements, it is projected that 1.8 million neonates will die in 2030.[3] Innovations in neonatal care, particularly technologies that allow for early detection and intervention for major morbidities, hold great promise in helping to reduce current and projected neonatal mortality rates.

Multiparameter continuous physiological monitoring devices could be instrumental in identifying neonates at risk. We can then direct care provided for a neonate through automatic interpretations of vital signs that help identify critical events and determine if treatment is sufficient or insufficient, ultimately improving newborn outcomes.[4 5] These devices would be most useful in low-resource settings where the need for such technologies is greatest. While continuous physiological monitoring is standard of care in high-resource settings for those who require it, the devices are expensive and require specialized training to operate, making them unsuitable for application in low-resource settings. To address these barriers, it is necessary to explore how these technologies can be adapted and/or optimized for use in low-resource settings. Ideally, the devices should be low cost, operator-independent, noninvasive and highly efficient in diagnostic performance and operator workload. This requires development of a robust testing platform that appropriately mimics

For peer review only - http://bmjopen.bmj.com/site/about/guidelines.xhtml

conditions common in African newborn or neonatal intensive care units that would allow these type of technologies to be evaluated for feasibility and performance.

The Evaluation of Technologies for Neonates in Africa (ETNA) project was conceived with the goal of advancing and supporting development, as well as evaluation, of select devices for use in neonates in low-resource settings. By establishing a testing platform in an African site, and working collaboratively with partners with expertise in device development and evaluation and neonatal and child health, the project seeks to boost development and optimization of promising newborn care devices that could be applied in low-resource settings in Africa. We acknowledge the many challenges involved in implementing such devices in low-resource settings (e.g., electricity and internet access, behavior change communication, etc.), and the need to consider these challenges carefully prior to introduction. The purpose of this initial research is to produce evidence regarding the performance of two existing noninvasive, multiparameter, continuous physiological monitoring devices developed by device developers, EarlySense and Sibel, to accurately and reliably measure vital signs in neonates (when compared to verified reference devices) and to assess the feasibility, usability and acceptability of these devices for use in neonates in a low-resource setting in Africa.

METHODS AND ANALYSIS

Study design and setting

The primary objectives of this prospective, observational, facility-based research are: 1) to assess agreement between repeat observations by the investigational device and the

For peer review only - http://bmjopen.bmj.com/site/about/guidelines.xhtml

reference device for each relevant measurement parameter of interest based on *a priori*-determined accuracy threshold among neonates; 2) to compare clinical event detection performance between the investigational device and the reference device; and 3) to determine whether the investigational device is feasible, usable and acceptable to hospital administrators, healthcare providers and caregivers of neonates. Secondary objectives include: 1) assessing diagnostic performance for each relevant measurement parameter of interest based on sensitivity, specificity, positive predictive value, and negative predictive value compared to the reference device; 2) determining the downtime performance of the investigational device; 3) determining the alarm rate (events/hour) and the number of true/false alarms of the investigational device compared to the reference device; 4) determining the delay time between the investigational device and the reference device in true events; and 5) determining the number of adverse device effects (ADEs) and serious adverse events (SAEs) during use of the investigational device.

Beginning in June 2019 and anticipated to last approximately 18 months in Nairobi, Kenya, this research consists of two phases: 1) a verification and accuracy evaluation phase conducted at Aga Khan University – Nairobi (AKU-N), a private, not-for-profit university teaching hospital with a neonatal intensive care and high dependency units; and 2) a clinical feasibility phase conducted at Pumwani Maternity Hospital (PMH), the largest referral maternity hospital in sub-Saharan Africa with no neonatal intensive care or high dependency units.

Study participants

Up to 500 neonates, corrected age of ≤28 days admitted for routine observation and care at AKU-N and PMH are recruited by trained study staff during routine intake and screening procedures. To avoid potential selection bias, neonates are screened for enrollment in a sequential manner, as much as possible. Trained study staff assess the neonate for all

For peer review only

For peer review only - http://bmjopen.bmj.com/site/about/guidelines.xhtml

inclusion and exclusion criteria (Table 1). Final eligibility determination is dependent on the results of the medical history, clinical examination, appropriate understanding of the study by the caregiver, and completion of the written informed consent process. A neonate may be enrolled to the study more than once as long as they meet the eligibility criteria and the caregiver(s) is willing to have the neonate participate.

For the feasibility, usability and acceptability assessment, hospital administrators and study healthcare providers are enrolled if they are 18 years or older, involved in or aware of the ETNA study, and have provided written informed consent. Caregivers may be enrolled if they are 18 years or older, have a neonate enrolled in the study, and are willing to participate in a 30-minute in-depth interview as well as direct observation while their neonate is on or attached to the investigational device(s).

Investigational devices

Developed by Israeli-based EarlySense since 2009, the Insight system, released in 2016, is a contact-free monitoring system comprised of a small piezoelectric sensor pad that can be placed under the patient's mattress, and is designed to measure and record a patient's heart rate (HR), respiratory rate (RR), motion, and sleep status.[6] Information from the sensor pad, in combination with EarlySense's artificial intelligence analytics, is transmitted to a monitor to provide alert indications and vital sign trends to healthcare providers so that they can monitor changes in a patient's condition. Currently in use in hospitals, rehabilitative centers, and nursing homes to measure vital signs in adults and children above 10 kg, the device is modified for use in neonates as part of this study. The adult device received regulatory approval from the United States Federal Drug Administration (FDA) and has a Conformité Européene (CE) mark for continuous and contactless measurement of HR,

For peer review only - http://bmjopen.bmj.com/site/about/guidelines.xhtml

RR and motion. No adverse events (AEs) related to the system have been reported during 10 years of monitoring.

Developed in 2019, the advanced neonatal epidermal (ANNE) system from Sibel, a technology company spun out from the Center of Bio-Integrated Electronics at Northwestern University in the United States, is a system of two time-linked soft and flexible sensors designed to measure and monitor vital signs including HR, RR, oxygen saturation (SpO_2), and skin temperature in neonates.[7] The chest sensor couples to the skin via a hypo-allergenic, biocompatible hydrogel adhesive optimized for reduced peel force upon removal, and the limb unit couples via a latex-free soft fabric wrap adaptable to a range of foot sizes and anatomies. Information from the sensors are wirelessly transmitted to a monitor or mobile device via encrypted Bluetooth™ for real-time streaming from a customized mobile software application as well as onboard memory storage on the sensors themselves. The device has been validated in more than 50 neonates in a neonatal care unit without AEs.

For peer review only

Reference devices

We are employing the Masimo Rad-97™ and the Spengler Tempo Easy Bleu devices as our reference devices for this study. The Masimo Rad-97™ provides continuous physiological monitoring of HR, RR, SpO2, and capnography. The Spengler Tempo Easy Bleu non-contact infrared thermometer predicts core body temperature from the temporal artery temperature.

Study procedures

Following completion of screening for eligibility, a study comprehension checklist, and written informed consent, study staff perform procedures (Appendix I: Schedule of study

For peer review only - http://bmjopen.bmj.com/site/about/guidelines.xhtml

procedures and evaluations) according to the most recently approved version of the protocol (current version 1.1, June 18, 2019). Enrolled neonates are assigned a participant identification number; information is collected on socio-demographic characteristics, current clinical status, medical history, medications; and a physical examination is performed.

Prior to initiating the accuracy evaluation of each investigational device, verification of the reference devices, Masimo Rad-97™ and Tempo Easy Bleu, is undertaken at AKU-N to ensure they are robustly functional and to confirm their within subject repeatability and accuracy compared to standard clinical measurements (e.g., manual, bedside electrocardiography) for the relevant parameters of interest. Neonates enrolled during reference device verification continue to receive local standard of care while being observed intermittently for vital signs collection for a minimum of 1 hour using the Masimo Rad-97™ and intermittent measurements with the Tempo Easy Bleu. Observations may include video recordings of the neonate and the Masimo Rad-97™reference device monitor for later review to facilitate manual count observations. The reference device measurements will be compared to manual measurements, clinical monitor observations, and video-assisted observations. Reliability information gathered from the reference devices is utilized to determine specific Go/No Go criteria for each parameter and each investigational device. Further evaluation of each investigational device only proceeds should these criteria be met.

Enrollment in the accuracy evaluation of the investigational devices, EarlySense Insight system and Sibel ANNE system, is initiated at AKU-N to formally assess their accuracy compared to the verified reference device using repeated observations. Enrolled neonates continue to receive local standard of care while having vital signs collected from the

For peer review only

reference device as well as one or both of the investigational devices. Placement of the investigational and reference devices is done in a manner so as not to interfere with the neonate's clinical care. Observations are collected for a minimum of 1 hour and potentially for the entire duration of their stay in the hospital. Observations may consist of videotaping and/or taking photos of the neonate during the observation period after obtaining informed consent from the caregiver. Any photos or videos takes are identified by patient identification number only and stored on a secure server until the analyses are completed and destroyed following analyses. During observation, clinical status and any activities are updated and recorded including type and duration of care activities (e.g., feeding, diaper changes, bathing, kangaroo mother care, etc.), clinical procedures, interventions, therapies, laboratory tests, medications, environmental features and exposures during hospitalization. The device placement, output, and signal quality are also monitored. In addition, the neonates are assessed for any safety issues. Agreement between the investigational and reference devices is determined using *a priori*-defined accuracy thresholds. Thresholds are determined largely based on repeated within and between subject observations during verification of the reference devices. This is complemented by previously published international standards where available, and clinical expert consensus opinion as needed. Two *a priori*-determined thresholds are determined: one lower threshold to allow the device developer to optimize the device for retesting, and a second higher threshold to allow the device to move on to the clinical feasibility phase of testing. A maximum of 5 rounds of testing and retesting are permitted for each investigational device. Each round of testing or retesting consists of using a cohort of 20 neonates. Should the lower threshold not be reached for at least one parameter, no further testing of the investigational device is performed. Thus, information collected during the accuracy evaluation along with the *a*

For peer review only

For peer review only - http://bmjopen.bmj.com/site/about/guidelines.xhtml

priori–determined Go/No Go criteria established during verification of the reference devices define which, if any, of the investigational devices moves forward with additional rounds of testing or into the clinical feasibility phase at PMH.

An investigational device advances to the clinical feasibility phase once the agreement for the measurement parameters of interest exceed the higher accuracy threshold. Enrollment in the clinical feasibility phase of the investigational devices occurs at PMH in up to 120 enrolled neonates who receive local standard of care while being monitored with the reference device(s) and one or both of the investigational devices. Observations are collected for a minimum of 1 hour and involve measurement of vital signs via the investigational and reference devices and monitoring for any critical event (i.e., low or high HR, RR, or temperature or oxygen desaturation and apnea). Agreement between repeated observations from the investigational and reference devices as well as diagnostic performance in clinical event detection is evaluated. Additional performance metrics such as alarm rates, alarm delays and uptime\downtime are compared between the investigational and reference devices. Participation in the study does not interfere with or unnecessarily delay the clinical care of the neonates.

Throughout all phases of the research, the investigational devices are not used to inform clinical care. During the clinical feasibility phase, ETNA site study staff and hospital healthcare providers are blinded to the data collected from the investigational devices to prevent interference with clinical care. The study site investigators are responsible for close safety monitoring of all participating neonates, including assessing for and reporting adverse device effects (e.g., erythema or edema at the investigational or reference device sensor site) and/or serious adverse events (i.e., any adverse device effect resulting in permanent

For peer review only

skin damage). Any adverse device effects or serious adverse events will be treated until resolution or stabilization, and may require removal of devices and withdrawal of the neonate from the study if necessary. If withdrawn by the study team, any enrolled neonate who completes at least one hour of monitoring will be included in the analysis and results.

Qualitative substudy

After written informed consent is received from the study participants, a mixed methods evaluation and data collection through audio-recorded semi-structured in-depth interviews and direct observations are conducted by trained qualitative study staff to assess the feasibility, usability, and acceptability of the investigational devices for monitoring of neonates in an African-setting. Questions around technology use, experience with continuous monitoring devices, and specific to each investigational and reference device will be asked and their use observed. All hospital administrators and study healthcare providers may be involved in this portion of the study. Caregivers with a neonate enrolled in the study may also be asked if they would like to participate in the qualitative portion of the study.

Sample size

A total of up to 500 neonates are enrolled. For the verification of the reference devices at AKU-N, up to 30 neonates are enrolled. Once this initial testing and data collection of the reference devices are complete, for the accuracy evaluation phase at AKU-N, up to 120 neonates per investigational device are enrolled. Sample size estimates for the verification of the reference devices and the accuracy evaluation phase are based on the confidence intervals (CIs) desired for the limits of agreement. Sample sizes of 100-200 typically provide tight CIs. A sample of 20 neonates with 10 replications per neonate per device per round of

For peer review only - http://bmjopen.bmj.com/site/about/guidelines.xhtml

testing provides limits of agreement with 95% CIs +/- 0.24, calculated as 1.96*sqrt(3/(20*10)), times the standard deviation of the paired differences. The paired differences are from the reference device and manual measurements obtained during verification of the reference device, and from the reference device and investigational device measurements obtained during the accuracy evaluation phase. For the clinical feasibility phase at PMH, up to 120 neonates per investigational device are enrolled. The sample sizes for each phase have been selected to maximize the amount of information collected within the confines of the available resources.

For the feasibility, usability, and acceptability assessment, the total sample size includes all hospital administrators and study healthcare providers willing to participate and provide consent as well as up to 30 caregivers willing to participate and provide consent study at each site.

Data collection and quality assurance

Quantitative study data is collected by clinical study staff using designated source documents as well as electronic or paper-based case report forms. Data is stored and managed by a database developed via Research Electronic Data Capture (REDCap), a secure web application. Continuous physiological data and event data are recorded from the investigational and reference devices at least once a second. All electronic data are collected wirelessly or via a wired connection from the investigational and reference devices to a study laptop using custom software applications. Qualitative study data is collected using paper-based forms and audio recordings which are subsequently transcribed for analysis.

For peer review only - http://bmjopen.bmj.com/site/about/guidelines.xhtml

Clinical research data, including data collected from the investigational and reference devices, are maintained through a combination of secure electronic data management system and physical files with restricted access to ensure confidentiality. Two distinct study databases are maintained separately: the primary study database and a database with participating neonate’s personally identifiable information. To ensure accuracy and completeness, data is routinely reviewed by the investigators through quality assurance reviews, audits, and evaluation of the study safety and progress. Guideline for Good Clinical Practice (GCP)/ ISO 14155 compliance is followed to ensure accurate, reliable, and consistent data collection.

For peer review only

Data management

Primary data management activities, which include de-identified investigational and reference device data transfer using end-to-end encryption with two-factor authentication, data entry and validation, data cleaning, database quality control, and disaster recovery plans are undertaken at the study site and are overseen by the on-site data manager. Data review and analysis, oversight and preparation of final study database is performed by the investigators in collaboration with the study site. Data are maintained and stored securely in databases hosted at the study site throughout the study and for at least 5 years after study closure. All data management activities are in compliance with International Council on Harmonization (ICH) GCP E6, sponsor organization, and institutional requirements for the protection of children and confidentiality of personal and health information.

Outcomes

For peer review only - http://bmjopen.bmj.com/site/about/guidelines.xhtml

We hypothesize that the investigational device is accurate and reliable compared to the reference device for each relevant measurement parameter of interest among neonates and is feasible, usable and acceptable for use in neonates in low-resource settings. The primary endpoint and secondary endpoints are detailed in Table 2.

Statistical analyses

Every second of data is automatically graded as optimal, acceptable and unacceptable based on predefined rules for each device and each measurement parameter of interest according to the quality of the data for each measurement parameter of interest. The Masimo Rad-97™ provides a signal quality index that is used to determine data quality for HR and SpO2. A custom algorithm has been produced to determine the capnography signal quality index. Each of the investigational devices also provides a signal quality index. The quality thresholds are determined following verification of the reference devices. All comparisons are performed from observations between two devices (or a single device during verification). At least 10 observations of 60 seconds of optimal quality data in each neonate, at least 5 minutes apart, are randomly selected for each measurement parameter of interest from the full recording. For the clinical feasibility phase, accuracy comparisons use optimal or acceptable data. At least 3 hours of recording to a maximum of 12 hours are used for the performance metrics such as alarm rates, alarm delays and uptime\downtime.

The repeatability of the reference device parameter estimates initially is assessed with the intraclass correlation coefficient (ICC). Additional training or standardization of procedures is performed to ensure at least good repeatability (ICC >0.7). This is followed by measuring agreement between the repeated reference observations and between the manual, clinical monitor and video-assisted methods and the reference observations using the methods

For peer review only

For peer review only - http://bmjopen.bmj.com/site/about/guidelines.xhtml

described by Bland and Altman for replicated observations.[8] The agreement is reported as a mean bias with 95% CIs and 95% limits of agreement. Graphical representation of the data is assessed with agreement plots, Clark error grids, and Polar plots to identify extreme outliers and significant data trends.

In the accuracy evaluation, the root mean square difference (RMSD) and ICC are calculated for each measurement parameter of interest to compare the multiple repeated observations between the investigational and reference devices. The agreement between each investigational device and reference device(s) is then calculated using the methods described by Bland and Altman for replicated observations. The agreement is reported as a mean bias with 95% CIs and 95% limits of agreement. Graphical representation of the data is be assessed with agreement plots, Clark error grids, and Polar plots to identify extreme outliers, impact on clinical decisions, and significant data trends. An *a priori*–defined accuracy margin for agreement is used as a threshold value to allow for decisions regarding proceeding to additional testing.

In the clinical feasibility phase, agreement between each investigational device and reference device(s) is assessed as in the accuracy evaluation phase. Event detection rates, alarm rates, alarm delays and uptime/ downtime are summarized with means, medians, standard deviations and intra-quartile ranges as appropriate. Summaries of sensitivity, specificity, positive predictive values and negative predictive values comparing each measurement parameter of interest in the investigational device(s) to the reference device(s) are produced. Comparisons of binary events are assessed using Cohen’s weighted Kappa and McNemar’s test. The non-inferiority of alarm rates, alarm delays and uptime/ downtime are evaluated based on pre-specified thresholds.

For peer review only

For peer review only - http://bmjopen.bmj.com/site/about/guidelines.xhtml

Qualitative data are collected through in-depth interviews and/or semi-structured questionnaires and analyzed to assess feasibility, usability, and acceptability of the investigational devices among hospital administrators and healthcare providers, and acceptability among caregivers of enrolled neonates. Questions that explore familiarity, knowledge, perceptions, attitudes and behaviors regarding the devices are included. The qualitative data is in narrative format and the results are descriptive. The questionnaires are coded and analyzed using a codebook with identified themes, including feasibility of using each investigational device, barriers and facilitators to use, and perceived value. Qualitative data analysis software is used to organize, code, and analyze the qualitative data in an iterative process. The study team starts by identifying an initial set of codes and themes based on the categories from the interview guides. During the coding process, attention is paid to identifying emergent issues and themes that are added to the codebook and included in the analysis. Responses from the interviews are coded and discrepancies discussed and resolved for the final analysis and theme identification.

ETHICS AND DISSEMINATION

Ethical approvals and consent

The study is conducted in accordance with the ICH GCP and the Declaration of Helsinki 2008. The protocol and other relevant study documents study were approved by the Western Institutional Review Board 20191102 (Puyallup, Washington, United States of America), and the Aga Khan University Nairobi Research Ethics Committee 2019/REC-02 (v2)(Nairobi, Kenya). Written informed consent is obtained in the local language by trained study staff

For peer review only

For peer review only - http://bmjopen.bmj.com/site/about/guidelines.xhtml

from all eligible neonate's caregivers and for the qualitative substudy, from participating hospital administrators, healthcare providers, and caregivers prior to enrollment. Potential participants will have adequate time to ask questions and a comprehension checklist will be administered to ensure participant understanding.

Possible risks

Caregivers may feel compelled to enroll in the study in order to receive care for their neonate within a research setting, which may be perceived as of a higher quality than the standard of care. In order to minimize the risk of coercion, during the informed consent process, study staff emphasize that the neonate will receive the required medical care whether enrolled in the study or not. Other potential risks to study participation may include those associated with the placement and attachment of the investigational and reference devices, and delayed medical management. Study staff are trained in the appropriate placement of investigational and reference devices' sensors to minimize discomfort to the neonates as well as to avoid interference with any assessment, treatment, or intervention necessary for clinical care. There is a potential risk of skin irritation with the ANNE sensor system and neonates will be closely monitored and treated for any AEs. Study staff are also trained in integrating study procedures with clinical care and to always prioritize clinical care above study procedures. Extreme care is taken to ensure that no necessary treatment is delayed to accommodate study procedures.

Dissemination

We plan to disseminate study results in peer-reviewed journals and international conferences, targeting those involved in the clinical care of neonates in low-resource

For peer review only - http://bmjopen.bmj.com/site/about/guidelines.xhtml

settings as well as those who develop and advise on policies and guidelines in those settings.

The trial is registered with ClinicalTrials.gov (registration number NCT03920761).

Efforts towards rigorous protocol

Dedicated study staff trained in GCP, operation, use and maintenance of the investigational and reference devices, and study-specific procedures follow neonates enrolled in the trial to assure the protocol and standard operating procedures are followed and data are accurately collected. Standardized study-specific training, supervision, and oversight are undertaken to ensure quality, consistency, and harmonized trial procedures and implementation. Regular monitoring is provided by the co-investigators to assess compliance with human subjects and other research regulations and guidelines, adherence to the study protocol and procedures, and quality and accuracy of data collected.

Limitations and bias

Limitations to this study and potential sources of bias include the sampling strategy, the uncertainty inherent in the measurements from the reference devices, the limited standardization of time of day of recording, and the inability to control the conditions and standardize the context. Because there is a large variation in the various ages, weights, sizes, disease states, clinical presentations, interventions received, and conditions of the participating neonates, it is not possible to control for all these variables. Likewise, the environment cannot be controlled, does not allow for complete standardization, and may introduce additional sources of bias. These limitations may also be viewed as strengths.

DECLARATIONS

Authors' contributions ASG, EN, WM and MA designed the study and wrote the protocol.

For peer review only

For peer review only - http://bmjopen.bmj.com/site/about/guidelines.xhtml

RO, MW, GZ, RK, and SX reviewed and provided critical input to the study design and protocol. ASG wrote the first draft of the manuscript, and EN and MA provided additional input. All authors worked collaboratively, reviewed the manuscript, and made the decision to submit the final manuscript for publication.

Acknowledgments We would like to thank Dustin Dunsmuir who wrote the IAP logger application used to collect the high resolution data from the reference device.

Funding This work is supported by grants from the Bill & Melinda Gates Foundation (OPP1203136) and the Save the Children Innovation Council. The authors had final responsibility for the decision to submit this manuscript for publication.

Competing interests RK is employed by EarlySense Ltd. and SX is employed by Sibel Inc. All other authors declare that they have no competing interests.

Ethics approval and consent to participate The study was approved by the Western Institutional Review Board 20191102 (Puyallup, Washington, United States of America) and the Aga Khan University Nairobi Research Ethics Committee2019/REC-02(v2) (Nairobi, Kenya). Written informed consent is obtained by trained study staff from all eligible children’s caregivers prior to enrollment.

Consent for publication Not applicable.

Patient and public involvement Patients and the public were not involved in the design of, recruitment to, or the conduct of the study.

Availability of data and materials Data will be made available on an open access platform after the publication of the main manuscripts. Processes will be developed to facilitate data sharing for scientific utilization in a collaborative manner.

For peer review only

For peer review only - http://bmjopen.bmj.com/site/about/guidelines.xhtml

TABLES AND FIGURE LEGENDS

Table 1. Eligibility criteria

**Eligibility criteria**	
Inclusion criteria	• Male or female neonate, corrected age of $\leq$28 days.<br>• Willingness and ability of neonate's caregiver to provide informed consent and to be available for follow-up for the planned duration of the study.
Exclusion criteria	• Receiving mechanical ventilation or continuous positive airway pressure.<br>• Skin abnormalities in the nasopharynx and/or oropharynx.<br>• Contraindication to application of skin sensors.<br>• Known arrhythmia.<br>• Presence of a congenital abnormality requiring major surgical intervention.<br>• Any medical or psychosocial condition or circumstance that, in the opinion of the investigators, would interfere with the conduct of the study or for which study participation might jeopardize the neonate's health.

For peer review only

453 **Table 2. Study endpoints**

**Primary endpoints**
• Agreement of the relevant measurement parameters of interest between the investigational device and the reference device at each observation.<br>• Agreement of clinical event detection between the investigational device and the reference device at each observation.<br>• Feasibility, usability and acceptability of the investigational device among hospital administrators and healthcare providers.<br>• Acceptability of the investigational device among caregivers.
**Secondary endpoints**
• Diagnostic performance of the investigational device to appropriately identify the following critical events:<br>○ Low heart rate<br>○ High heart rate<br>○ Low respiratory rate<br>○ High respiratory rate<br>○ Oxygen desaturation<br>○ Apnea<br>○ Low temperature<br>○ High temperature<br>• Downtime duration of the investigational device.<br>• Alarm rate (events/hour and ratio of false positives to missed critical events of the investigational device's alarms compared to the reference device's alarms.

For peer review only - http://bmjopen.bmj.com/site/about/guidelines.xhtml

- Response time of the investigational device's alarms compared to the reference device's alarms for critical events.
- Proportion of neonates with adverse device effects and serious adverse events resulting in skin damage.

References

1. United Nations Inter-agency Group for Child Mortality Estimation (UN IGME), 'Levels & Trends in Child Mortality: Report 2018, Estimates developed by the United Nations Inter-agency Group for Child Mortality Estimation' United Nations Children's Fund. New York, 2018.
2. Sustainable Development Goals Knowledge Platform. Division for Sustainable Development Goals.[website]. New York, NY: United Nations Headquarters. Date Accessed: September 22, 2019. https://sustainabledevelopment.un.org/sdg3.
3. Hug L, Alexander M, You D, et al. National, regional, and global levels and trends in neonatal mortality between 1990 and 2017, with scenario-based projections to 2030: a systematic analysis. *Lancet Glob Health* 2019;7(6):e710-e20. doi: 10.1016/S2214-109X(19)30163-9
4. Zhu Z, Liu T, Li G, et al. Wearable sensor systems for infants. *Sensors (Basel)* 2015;15(2):3721-49. doi: 10.3390/s150203721
5. Sahni R. Continuous noninvasive monitoring in the neonatal ICU. *Curr Opin Pediatr* 2017;29(2):141-48. doi: 10.1097/MOP.0000000000000459
6. EarlySense InSight User Guide - USA version, Doc. No. MK-3602-IS-US, Revision 3, 2017.
7. ANNE System Product Requirements Document, SH-PRD-Rev 2, February 11, 2019.
8. Bland JM, Altman DG. Measuring agreement in method comparison studies. *Stat Methods Med Res* 1999;8(2):135-60. doi: 10.1177/096228029900800204

For peer review only - http://bmjopen.bmj.com/site/about/guidelines.xhtml

Appendix I: Schedule of study procedures and evaluations

Activity	Screening	Enrollment	Observation	Discharge
Eligibility assessment	X			
Informed consent and comprehension checklist	X			
Assign participant ID	X			
Demographics	X	X		
Medical history		X	X	X
Maternal pregnancy history		X		
Medications use		X		
Placement of reference and/or investigational device(s)		X	X	
Collection of vital signs		X	X	X
Video tape recording and/or photographs			X	

For peer review only

Track clinical care and non-study activities			X	X
Safety assessment			X	
End of study questions				X
Removal of investigational and/or reference device(s)				X

2

For peer review only - http://bmjopen.bmj.com/site/about/guidelines.xhtml
